# Female Behaviour Drives Expression and Evolution of Gustatory Receptors in Butterflies

**DOI:** 10.1371/journal.pgen.1003620

**Published:** 2013-07-11

**Authors:** Adriana D. Briscoe, Aide Macias-Muñoz, Krzysztof M. Kozak, James R. Walters, Furong Yuan, Gabriel A. Jamie, Simon H. Martin, Kanchon K. Dasmahapatra, Laura C. Ferguson, James Mallet, Emmanuelle Jacquin-Joly, Chris D. Jiggins

**Affiliations:** 1 Department of Ecology and Evolutionary Biology, University of California, Irvine, California, United States of America; 2 Department of Zoology, University of Cambridge, Cambridge, United Kingdom; 3 Department of Biology, Stanford University, Palo Alto, California, United States of America; 4 Department of Biology, University of York, York, United Kingdom; 5 Department of Zoology, University of Oxford, Oxford, United Kingdom; 6 Department of Organismic and Evolutionary Biology, Harvard University, Cambridge, Massachusetts, United States of America; 7 Department of Genetics, Evolution and Environment, University College London, London, United Kingdom; 8 INRA, UMR 1272 INRA-UPMC Physiologie de l'Insecte: Signalisation et Communication, Versailles, France; University of Michigan, United States of America

## Abstract

Secondary plant compounds are strong deterrents of insect oviposition and feeding, but may also be attractants for specialist herbivores. These insect-plant interactions are mediated by insect gustatory receptors (*Grs*) and olfactory receptors (*Ors*). An analysis of the reference genome of the butterfly *Heliconius melpomene*, which feeds on passion-flower vines (*Passiflora* spp.), together with whole-genome sequencing within the species and across the *Heliconius* phylogeny has permitted an unprecedented opportunity to study the patterns of gene duplication and copy-number variation (CNV) among these key sensory genes. We report *in silico* gene predictions of 73 *Gr* genes in the *H. melpomene* reference genome, including putative CO_2_, sugar, sugar alcohol, fructose, and bitter receptors. The majority of these *Grs* are the result of gene duplications since *Heliconius* shared a common ancestor with the monarch butterfly or the silkmoth. Among *Grs* but not *Ors*, CNVs are more common within species in those gene lineages that have also duplicated over this evolutionary time-scale, suggesting ongoing rapid gene family evolution. Deep sequencing (∼1 billion reads) of transcriptomes from proboscis and labial palps, antennae, and legs of adult *H. melpomene* males and females indicates that 67 of the predicted 73 *Gr* genes and 67 of the 70 predicted *Or* genes are expressed in these three tissues. Intriguingly, we find that one-third of all *Grs* show female-biased gene expression (n = 26) and nearly all of these (n = 21) are *Heliconius*-specific *Grs*. In fact, a significant excess of *Grs* that are expressed in female legs but not male legs are the result of recent gene duplication. This difference in *Gr* gene expression diversity between the sexes is accompanied by a striking sexual dimorphism in the abundance of gustatory sensilla on the forelegs of *H. melpomene*, suggesting that female oviposition behaviour drives the evolution of new gustatory receptors in butterfly genomes.

## Author Summary

Insects and their chemically-defended hostplants engage in a co-evolutionary arms race but the genetic basis by which suitable host plants are identified by insects is poorly understood. Host plant specializations require specialized sensors by the insects to exploit novel ecological niches. Adult male and female *Heliconius* butterflies feed on nectar and, unusually for butterflies, on pollen from flowers while their larvae feed on the leaves of passion-flower vines. We have discovered–between sub-species of butterflies-fixed differences in copy-number variation among several putative sugar receptor genes that are located on different chromosomes, raising the possibility of local adaptation around the detection of sugars. We also show that the legs of adult female butterflies, which are used by females when selecting a host plant on which to lay their eggs, express more gustatory (taste) receptor genes than those of male butterflies. These female-biased taste receptors show a significantly higher level of gene duplication than a set of taste receptors expressed in both sexes. Sex-limited behaviour may therefore influence the long-term evolution of physiologically important gene families resulting in a strong genomic signature of ecological adaptation.

## Introduction

Nearly 50 years ago Ehrlich and Raven proposed that butterflies and their host-plants co-evolve [1]. Based on field observations of egg-laying in adult female butterflies, feeding behavior of caterpillars, and studies of systematics and taxonomy of plants and butterflies themselves, they outlined a scenario in which plant lineages evolved novel defensive compounds which then permitted their radiation into novel ecological space. In turn, insect taxa evolved resistance to those chemical defences, permitting the adaptive radiation of insects to exploit the new plant niche. Ehrlich and Raven's theory of an evolutionary arms-race between insects and plants drew primarily from an examination of butterfly species richness and host-plant specialization. It did not specify the sensory mechanisms or genetic loci mediating these adaptive plant-insect interactions.

**Figure 1:**
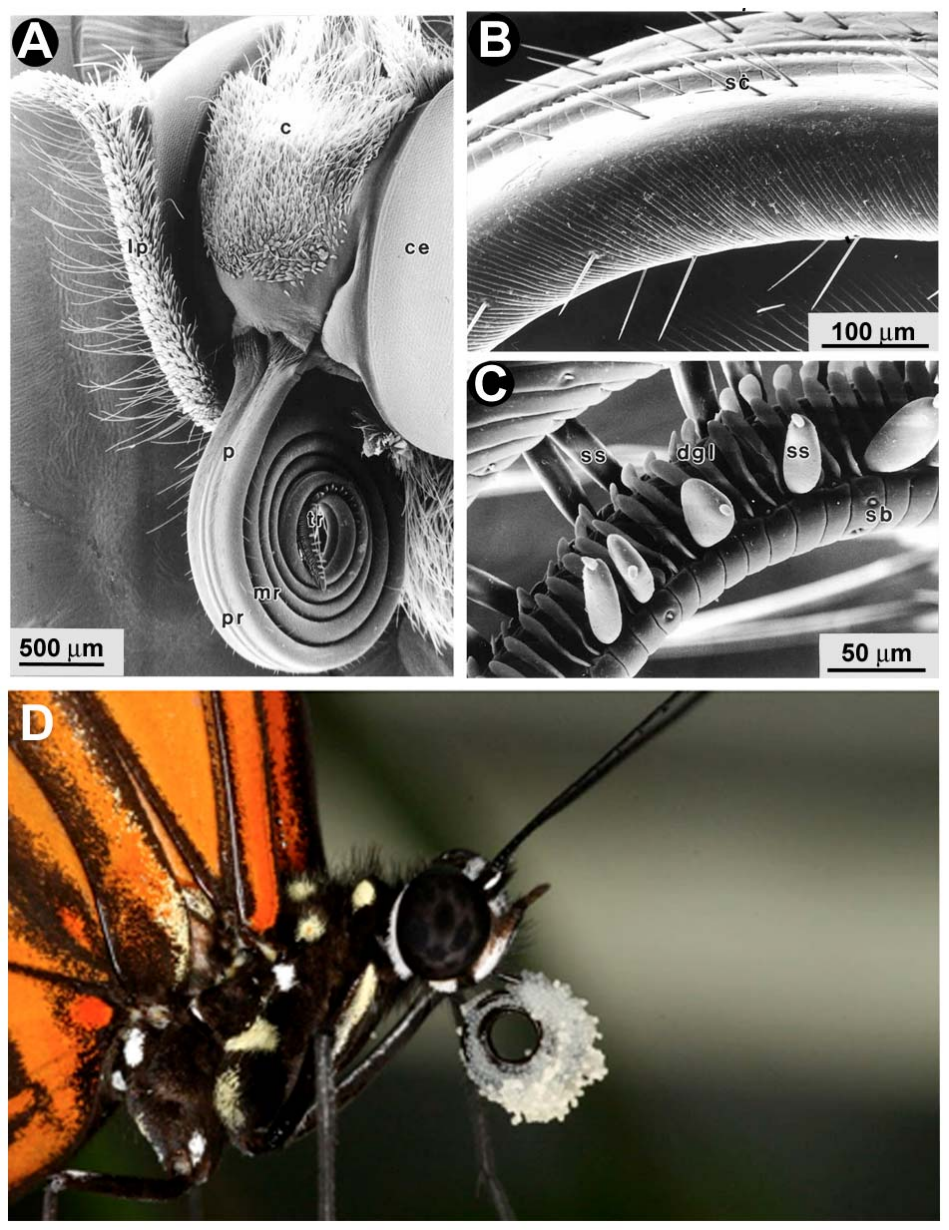
Scanning electron micrographs of the proboscis of *Heliconius* butterflies. (A) The labial palps (lp) and proboscis (p) of the *H. erato* head contain gustatory sensilla. (B) The proximal portion of the *H. melpomene* proboscis has hair-like *sensilla chaetica* (sc). (C) The tip portion of the proboscis has specialized ridges for pollen collection along with *sensilla styloconica* (ss). Reproduced with permission [9]. (D) *H. melpomene* with a pollen-load. c, clypeus, ce, compound eye; pr, proximal region; mr, mid region; tr, tip region; dgl, dorsal galeal linking structures; sb, blunt-tipped sensilla.

**Figure 2:**
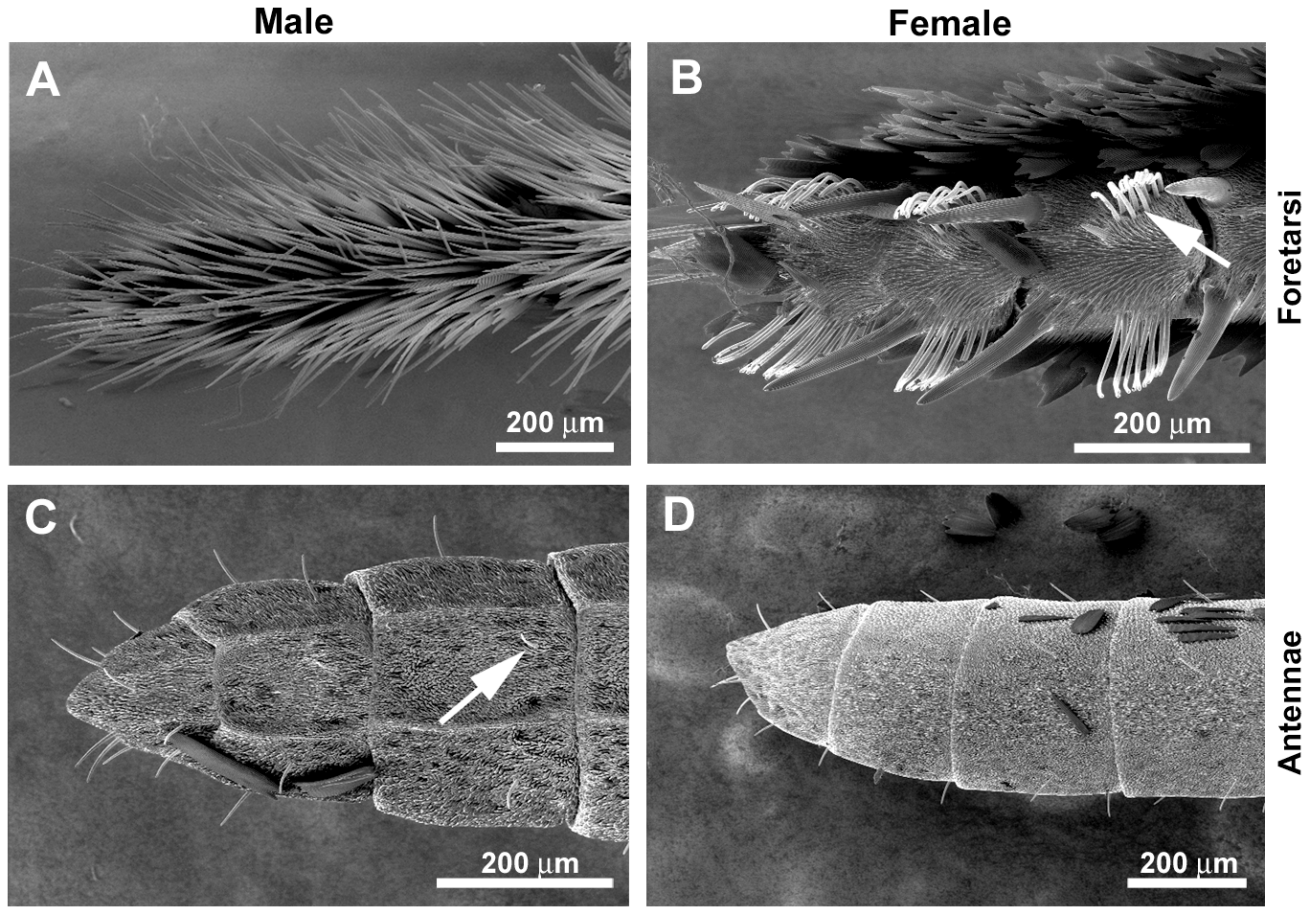
Sexual dimorphism in *H.* *melpomene* chemosensory tissues. Scanning electron micrographs of adult legs showing a sexual dimorphism in gustatory (trichoid) sensilla. Foreleg foretarsi of a male (A) and a female (B). Four pairs of clumped taste sensilla are each found associated with a pair of cuticular spines on each female foot (only three are shown). Arrow indicates a clump of taste sensilla. Antennae of an adult male (C) and a female (D) showing individual gustatory sensilla (arrow).

Insects possess gustatory hairs or contact chemosensilla derived from mechanosensory bristles, scattered along a variety of appendages [2]–[4]. In adult butterflies and moths, gustatory sensilla are found on the labial palps and proboscis (Figure 1), the legs (Figure 2A) [5], the antennae (Figure 2B) [6], [7], and the ovipositor [8], [9]. In adult *Heliconius charithonia* legs, the 5 tarsomeres of the male foreleg foretarsus are fused and lack chemosensory sensilla, while female foretarsi bear groups of trichoid sensilla (n = 70–90 sensilla/tarsus) associated with pairs of cuticular spines [10]. Each trichoid sensilla contains five receptor neurons. These sensilla are sensitive to compounds that may be broadly classified as phagostimulants (e.g., sugars and amino acids), which promote feeding behavior, or phagodeterrents (secondary plant compounds), which suppress it [11]; in adult females they may also modulate oviposition [12].

Genes for vision, taste and smell are likely to be crucial genomic loci underlying the spectacular diversity of butterfly-plant interactions. The availability of genomes for two butterfly species, the postman *Heliconius melpomene* (Nymphalidae) [13] and the monarch (*Danaus plexippus*) [14], as well as the silkmoth (*Bombyx mori*) [15], enables us to examine the evolutionary diversification of gustatory *(Gr)* and olfactory (*Or*) receptor genes that mediate insect-plant interactions. Each of these species feeds on hosts from different plant families. Silkmoth larvae feed on mulberry (*Morus* spp., Moraceae) and monarch larvae feed on milkweed (*Asclepias* spp., Apocynaceae). The larvae of *Heliconius* feed exclusively on passion flower vines, primarily in the genus *Passiflora* (Passifloraceae). In addition, adult *Heliconius* are notable for several derived traits such as augmented UV color vision [16], pollen feeding (Figure 1B) [17], [18], and the ability to sequester substances from their host plants that are toxic to vertebrate predators such as birds [19], [20].

In *Drosophila melanogaster*, the *Gr* gene family consists of 60 genes [21]–[24], several of which are alternatively spliced, yielding 68 predicted *Gr* transcripts [24]. One or more of these Gr proteins including possibly obligatory co-receptors [25]–[27] may be expressed in each gustatory receptor neuron [11]. Originally considered members of the G-protein-coupled receptor (GPCR) family, insect Grs have an inverted orientation in the membrane compared to the GPCR family of vertebrate *Grs* [28] and are part of the same superfamily as the insect *Ors* [21]. Signalling pathways for insect Grs may be both G-protein dependent [29], [30], [31] and G-protein independent [32]. For the vast majority of *Drosophila Grs* the specific compounds to which they are sensitive remain unknown. Nonetheless, several receptors for sugars [33]–[35], CO_2_ [26], [36], bitter substances [37]–[39] and plant-derived insecticides [25] have been identified in flies.

Knowledge of the *Gr* gene family for insects outside *Drosophila* is sparse and has primarily relied on the analyses of individual reference genomes. Expression studies are challenging, due to the very low expression of *Grs* in gustatory tissues [21], [23]. In addition, *Grs* and *Ors* typically have large introns, small exons and undergo fast sequence evolution, making their *in silico* identification using automated gene prediction algorithms from genomic sequences problematic. Thus, the large repertoire of *Grs* (and *Ors*) that have been examined in the reference genomes of the pea aphid [40], the honey bee [41], the red flour beetle *Tribolium castaneum* [42], the mosquitoes *Aedes aegypti* [43] and *Anopheles gambiae* [44], and several *Drosophila* spp. [45], [46] have required extensive manual curation. In Lepidoptera, a large insect group which includes ∼175,000 species, completely described *Gr* (and *Or*) gene models from genomes are rare and limited to *B. mori* [47], *D. plexippus* [14] and *H. melpomene* (*Grs*, this study; *Ors*, [13]). In other lepidopteran species, only fragmentary *Gr* data are available: five sequences in *Spodoptera littoralis* [48], three in *Heliothis virescens* [49], two in *Manduca sexta* [50], [51] and one in *Papilio xuthus* [52].

Adult females of each *Heliconius* species only lay eggs on a limited number of host plants [53], and therefore need to recognize different species from among the large and diverse Passifloraceae family, which also show a remarkable diversity of chemical defences [54]. The evolutionary arms race between *Heliconius* butterflies and their hosts led us to hypothesize that *Heliconius Grs* (and *Ors*) might be subject to rapid gene duplication and gene loss as well as copy-number variation (CNV). Recent work taking advantage of published *Drosophila* genomes has shown a relationship between host specialization and/or endemism and an increased rate of gene loss, as well as a positive relationship between genome size and gene duplication [46], [55]. Moreover, *Drosophila Grs* appear to be evolving under weaker purifying selection than *Ors* [55].

We previously used the reference genome sequence for *H. melpomene* to annotate three chemosensory gene families, encoding the chemosensory proteins (CSPs), the odorant-binding proteins (OBPs), and the olfactory receptors (Ors). This demonstrated a surprising diversity in these gene families. In particular there are more CSPs in the butterfly genomes than in any other insect genome sequenced to date [13]. We build on this work below by characterizing the *Gr* gene family in the reference *H. melpomene melpomene* genome and in two other lepidopteran species whose genomes have been sequenced, *B. mori* (Bombycidae) and *D. plexippus* (Nymphalidae), by performing *in silico* gene predictions and phylogenetic analysis. We then analyzed whole-genome sequences of twenty-seven individual butterflies, representing eleven species sampled across all major lineages of the *Heliconius* phylogeny and including sixteen individuals from two species, *H. melpomene* and its sister-species *H. cydno*. We also generated RNA-sequencing expression profiles of the proboscis and labial palps, antennae and legs of individual adult male and female butterflies of the sub-species *H. melpomene rosina* from Costa Rica (∼1 billion 100 bp reads). We used these data to address four major questions: Are different chemosensory modalities less prone to duplication and loss than others (e.g., taste vs. olfaction)? Is there evidence of lineage-specific differentiation of *Gr* (and *Or*) repertoires between genera, species and populations? What is the relationship between CNVs and the retention of paralogous genes over long-term evolutionary timescales? Are the life history differences between males and females reflected in the expression of *Grs* and *Ors* as well as in the retention of novel sensory genes in the genome?

We find higher turnover of the *Grs* than the *Ors* over longer evolutionary timescales, and evidence for both gene duplication and loss among a clade of intronless *Grs* between lepidopteran species and within the genus *Heliconius*. We also find for *H. melpomene* and its sister species, *H. cydno*, evidence of copy-number variation (CNVs) within their *Gr* and *Or* repertoires. Lastly, our RNA-sequencing suggests both tissue-specific and sex-specific differences in the diversity of expressed *Grs* and *Ors*, with female legs expressing a more diverse suite of *Grs* than male legs. Our data set revealing the expression of 67 of 73 predicted *Gr* genes and 67 of 70 predicted *Or* genes in adult *H. melpomene* butterflies is the most comprehensive profiling of these chemosensory gene families in Lepidoptera to date, and suggests how female host plant-seeking behaviour shapes the evolution of gustatory receptors in butterflies.

## Results

### Annotation of *Grs* in the reference genome of *H. melpomene*

In total, we manually annotated 86,870 bp of the *H. melpomene melpomene* reference genome (Table S1). Our 73 *Gr* gene models, consisted of 1–11 annotated exons, with the majority having three or four exons; six were intronless. We found genomic evidence (but not RNA-seq evidence) of possible alternative splicing of the last two exons of *HmGr18*, bringing the total number of predicted Grs to 74. Alternative splicing has not been previously described in the silkmoth *B. mori* [47], but is known to occur in most other insects examined, including *D. melanogaster*, *Anopheles gambiae*, *Aedes aegypti* and *T. castaneum* [24], [43], [44]. We also identified eleven new putative *Grs* in the monarch butterfly genome, *DpGr48-56*, *DpGr66* and *DpGr68* (Table S1) [14].

All but five of our gene models contained more than 330 encoded amino acids (AAs) while individual gene models ranged from 258–477 AAs. Several *Gr* genes contained internal stop codons (Table S1). In at least one case, we found RNA-seq evidence of an expressed pseudogene–*HmGr61*–with two in-frame stop codons. In other cases, the 5′ end of our assembled transcripts was not long enough to verify the internal stop codons in the genome assembly. The *Grs* are located on 33 distinct scaffolds, with 58 forming clusters of 2–8 genes on 18 scaffolds, distributed across 14 chromosomes.

### Gene duplication and loss in a clade of putative bitter receptors

**Figure 3:**
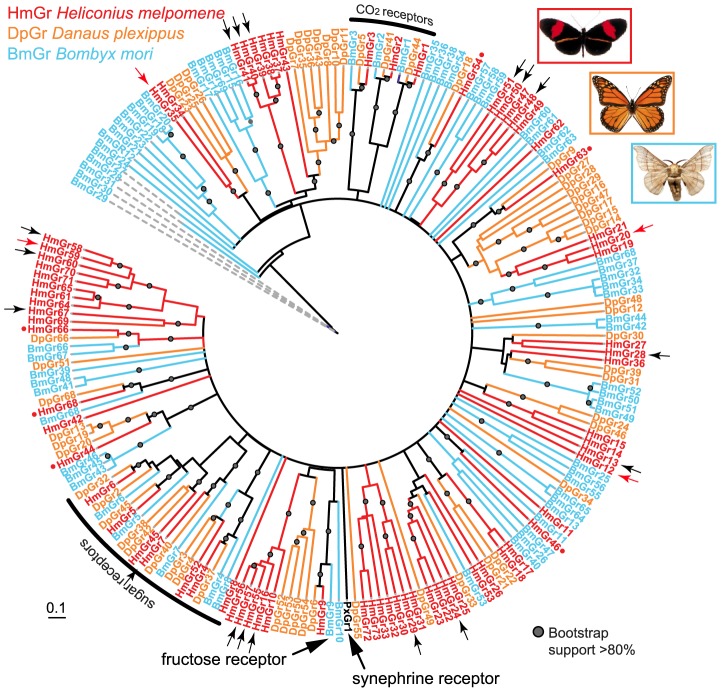
Phylogeny of the Grs identified in three lepidopteran genomes. A maximum likelihood analysis of amino acid sequences was performed. Bootstrap support is out of 500 replicates. Putative CO_2_ and fructose receptors show a conserved 1-to-1 orthologous relationship in each of the three lepidopteran genomes, while putative sugar receptors of the monarch butterfly have duplicated twice. By contrast, numerous butterfly- or moth-specific gene duplications are evident among the remaining *Grs*, which are hypothesized to be bitter receptors. Small red dots indicate single-copy *Heliconius Grs* classified as conserved genes in the analyses shown in Table 1 and Table 2. Small black arrows indicate female-specific *Grs* expressed in adult *H. melpomene* legs. Small red arrows indicate *Grs* expressed in adult *H. melpomene* proboscis only. Bar indicates branch lengths in proportion to amino acid substitutions/site. Synephrine and fructose receptors are described in [52] and [32]. Bm = *Bombyx mori*, Hm = *Heliconius melpomene*, Dp = *Danaus plexippus*, Px = *Papilio xuthus*.

**Figure 4:**
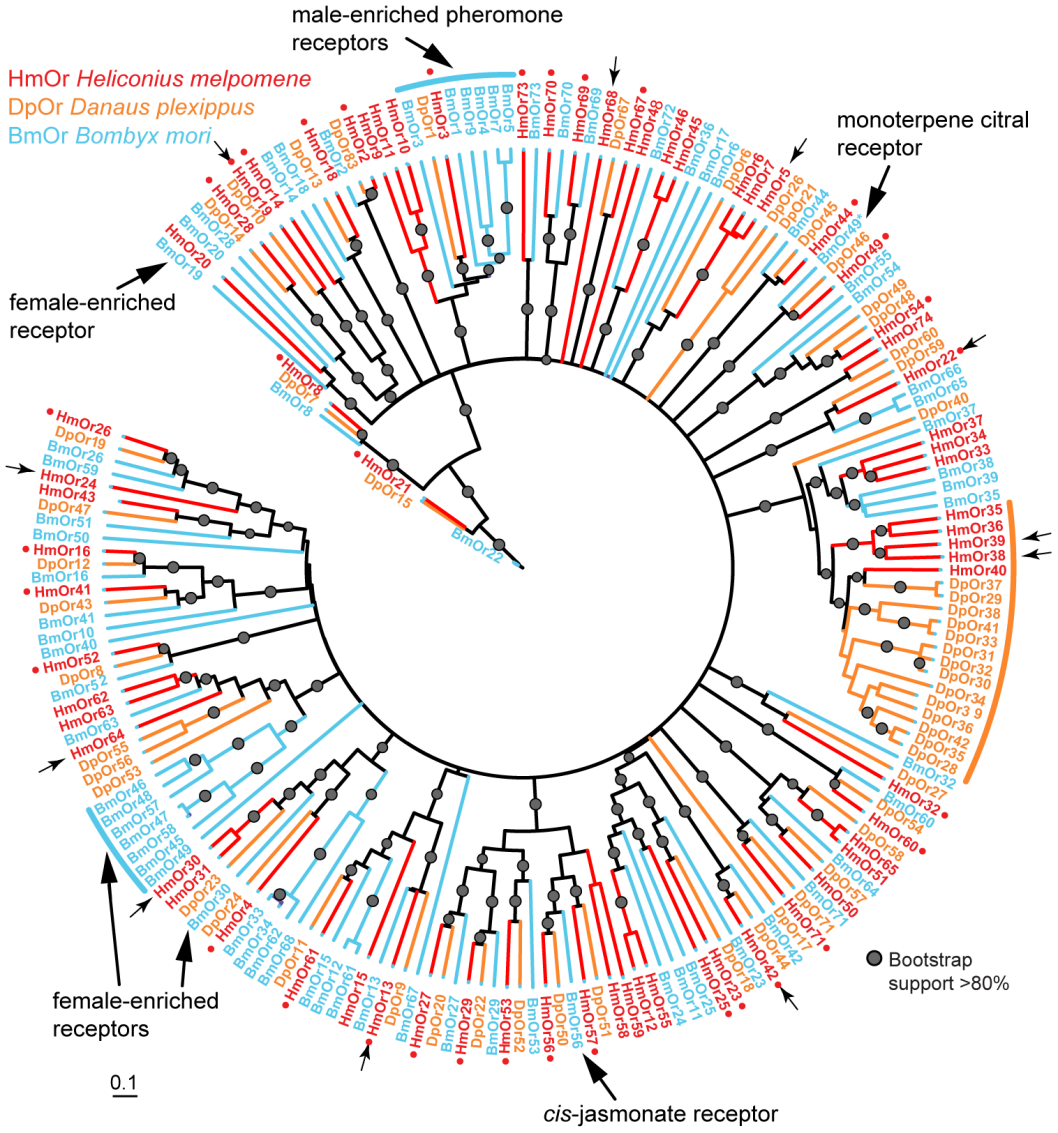
Phylogeny of the Ors identified in three lepidopteran genomes. A maximum likelihood analysis of amino acid sequences was performed. Bootstrap support is out of 500 replicates. Fewer lineage-specific duplications are evident among the *Ors* compared to the *Grs*, with the exception of one large butterfly-specific expansion (orange arc). Small red dots indicate single-copy *Heliconius Ors* classified as conserved genes in the analyses shown in Table 1 and Table 2. *Ors* that are enriched in male or female adult *B. mori* antennae (blue and black arcs) are described in [91]; *cis*-jasmonate and monoterpene citral receptors are described in [92] and [93]. Phylogenetic tree reconstruction details are given in [13]. Bar indicates branch lengths in proportion to amino acid substitutions/site. Small arrows indicate female-specific *Ors* expressed in adult *H. melpomene* legs. Bm = *Bombyx mori*, Hm = *Heliconius melpomene*, Dp = *Danaus plexippus*.

To study the patterns of gene duplication and loss more broadly across the Lepidoptera, we next examined the phylogenetic relationships of *Grs* from the three lepidopteran reference genomes [13]–[15]. Across the gene family phylogeny a large number of duplications among the putative ‘bitter’ gustatory receptors of *Heliconius* or *Danaus* have occurred, while the putative CO_2_ and sugar receptors are evolving more conservatively, with only single copies in the *H. melpomene* reference genome (see below)(black arcs, Figure 3). A majority (∼64%) of *Gr* genes found in the *H. melpomene* genome are the result of gene duplication since *Heliconius* shared a common ancestor with *Danaus* or *Bombyx*. This is in contrast to the more conserved pattern of evolution of the *Ors* (Figure 4) [13] where a majority (37 of 70 or 53%) of genes show a one-to-one orthologous relationship with either a gene in *Danaus*, in *Bombyx* or both.

**Figure 5:**
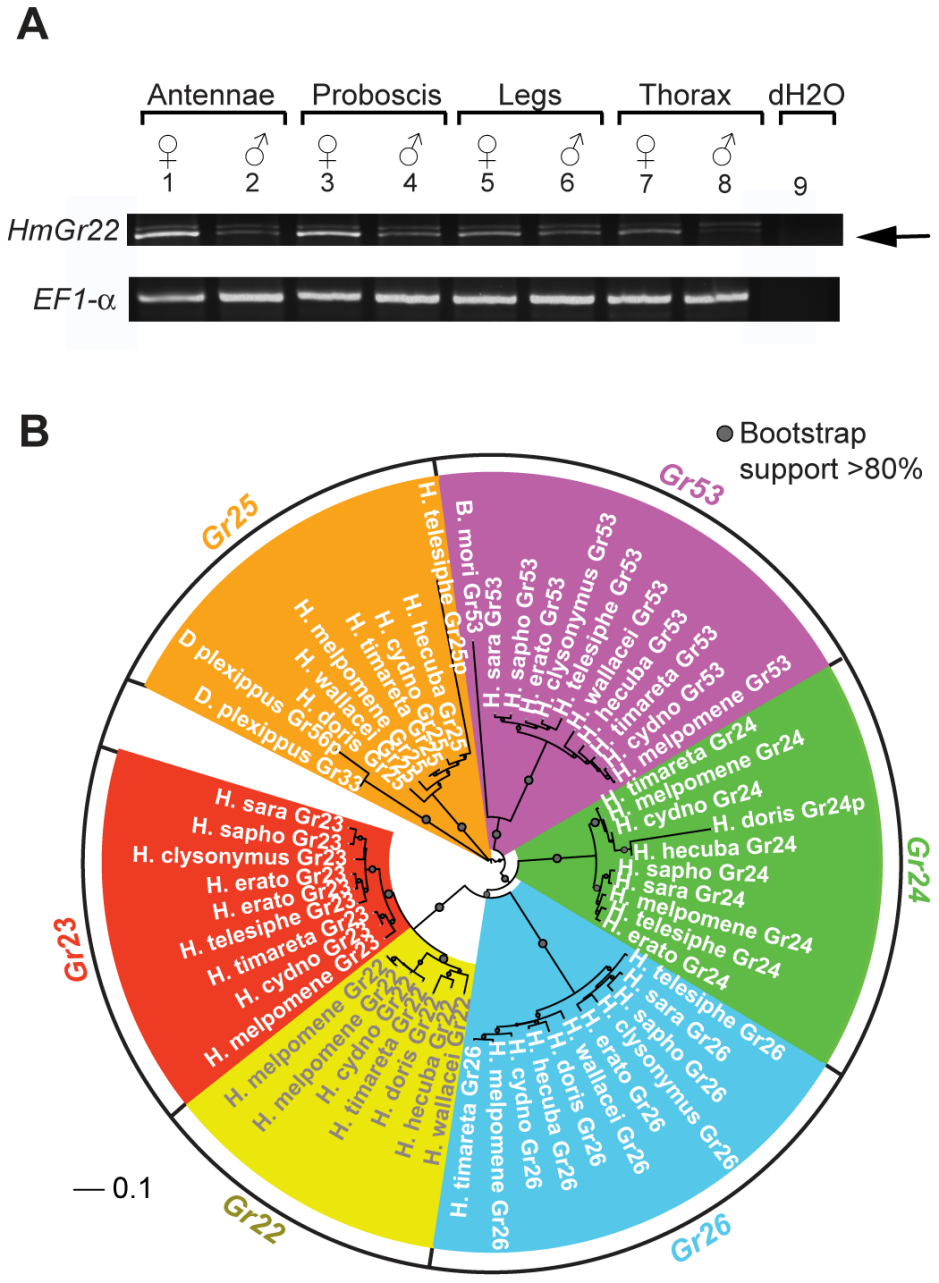
*HmGr22* expression in adults and intronless *Grs* from whole-genome sequence data across the *Heliconius* phylogeny. (A) Reverse-transcriptase PCR (RT-PCR) of adult *H. melpomene* tissues showing the expression of *HmGr22* and *elongation factor-1 alpha*. Two products are evident from the *Gr22* RT-PCR. The bottom RT-PCR product is *HmGr22* (arrow) and the top RT-PCR product is 18 s *rRNA*, which was verified by Sanger sequencing. (B) Neighbor-joining tree showing the phylogenetic relationship between the forty-six intact *Grs* and four pseudogenes identified in the 13 lepidopteran genomes. Bootstrap support is out of 500 bootstrap replicates. Pseudogene sequences are indicated by a ‘p’ after the gene name.

Within the genus *Heliconius* there is a great diversity of host plant preferences for different *Passiflora* species. To look at the relationship between gene duplication and loss over this shorter timescale, we focussed our efforts on a group of six intronless *Grs*, *HmGr22-26* and *Gr53*, because it is only feasible to identify single-exon genes with high confidence, given that the Illumina whole-genome sequencing approach leads to poorly assembled genomes (Table S2). These genes are also of interest as some members of this group are very highly expressed. Notably *HmGr22* is one of the most widely expressed genes in our adult *H. melpomene* transcriptomes, which was verified by reverse-transcriptase (RT)-PCR and sequencing of the PCR products (Figure 5A). In this regard *HmGr22* resembles another intronless *Gr*, the silkmoth gene *BmGr53*, which is expressed in adult male and female antennae and larval antennae, maxilla, labrum, mandible, labium, thoracic leg, proleg and gut [32]. The remaining five intronless *Grs* have much more limited domains of expression in adult *H. melpomene* (see below). We searched for these genes in *de novo* assemblies of whole-genome Illumina sequences from eleven species across the *Heliconius* phylogeny. We investigate whether, as in *Drosophila*, a high turnover in putative bitter receptors is observed in species with host plant specializations or in species which are endemic and thus smaller in effective population size [46].

**Figure 6:**
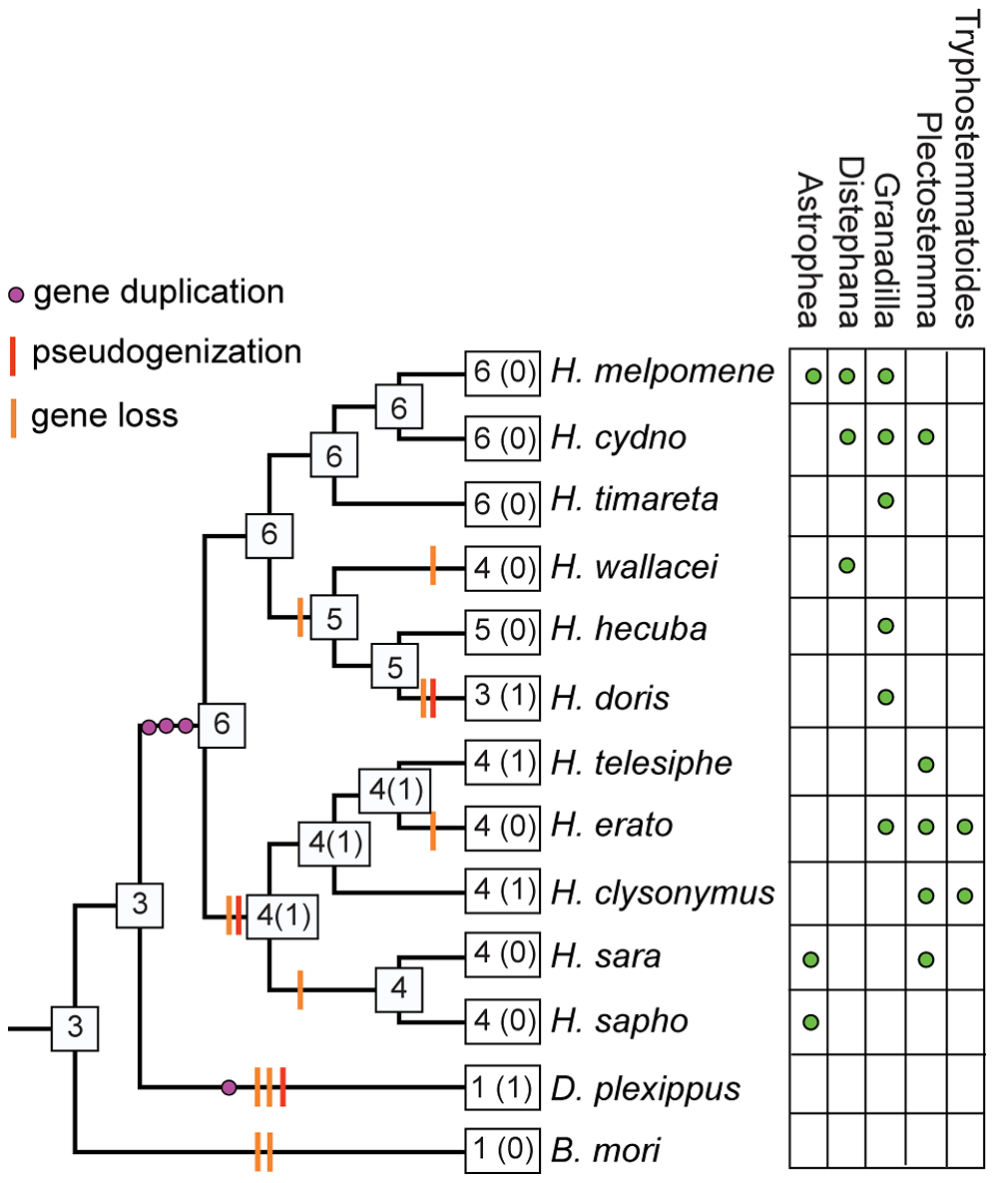
Inferred patterns of intronless *Gr* gene gain and loss across the genus *Heliconius*. Estimates of the number of *Gr* loci (number of pseudogenes is indicated in parentheses) on internal nodes of the lepidopteran phylogeny and gene gain (purple dots), gene loss (orange slashes) and pseudogenisation events (red slashes) on each branch. *Heliconius* phylogeny is based on Beltran et al. (2007) [90]. Reconciliation of gene trees onto the species tree was performed in Notung using maximum likelihood gene family trees. Primary *Passiflora* host plant subgenera (green dots) affiliated with each *Heliconius* species [53]. No clear relationship exists between the number of known *Passiflora* subgenera used and the number of intronless *Grs* in a species, which are presumed to be putative bitter receptors, but whose ligands are not yet identified. The woody vine specialist, *H. doris*, with the smallest effective population size, has the fewest intact intronless *Grs*.

Although patterns of host plant use are complex within the genus, some notable host-plant shifts have occurred, leading to the prediction that gene loss may have occurred along more specialized lineages [46]. For example, *H. doris* unlike many *Heliconius*, tends to feed on large woody *Passiflora* that can support their highly gregarious larvae [53]. It also probably has a smaller effective population size than most other *Heliconius* species. From the 11 species studied, we identified a total of 44 intact or nearly intact intronless *Grs*, as well as three intronless pseudogenes (Genbank Accession Nos. KC313949-KC313997)(Table S2 and S3). We also identified one intact intronless *Gr* each in monarch and silkmoth and one intronless *Gr* pseudogene in monarch. Phylogenetic analysis indicates that six intact intronless *Gr* genes were present at the base of the genus *Heliconius* while the intronless *Gr* pseudogene in monarch was the result of duplication since *Heliconius* and monarch shared a common ancestor (Figure 5B, Figure 6). Subsequent to the radiation of the genus *Heliconius*, there have been a number of gene losses. Whereas all members of the *melpomene* clade (*H. melpomene, H. cydno, H. timareta*) retained genomic copies of all six genes, members of the *erato* clade (*H. erato*, *H. clysonymus* and *H. telesiphe*) and *sara-sapho* clade (*H. sara* and *H. sapho*) have lost their copies of *Gr22* and *Gr25*. In addition, members of the so-called primitive clade (*H. wallacei*, *H. hecuba*, and *H. doris*) have lost *Gr23*, while *H. doris* and *H. wallacei* have apparently lost *Gr24* independently (Figure 6). The woody plant specialist, *H. doris*, has retained the fewest intronless *Grs*, apparently also having lost its copy of *Gr53*, a pattern mirrored by *Drosophila* host plant specialists [46]. We have, however, no direct evidence that the intronless *Grs* are in fact involved in host plant discrimination so the observed patterns of loss may be better explained by other variables such as effective population size.

### CNVs occur frequently among paralogous gustatory receptor genes

**Figure 7:**
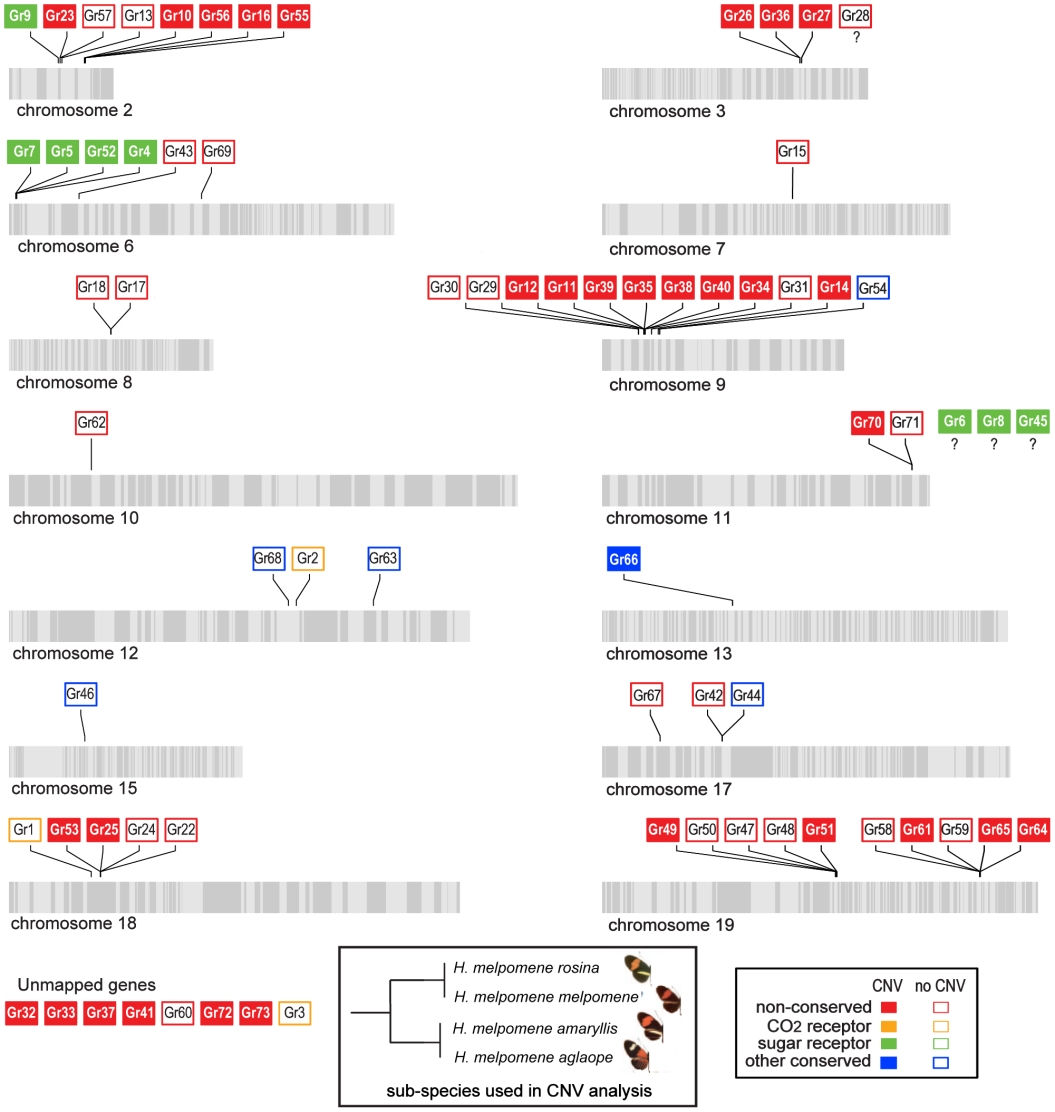
Copy-number variant (CNV) analysis of *Grs* in the *H.* *melpomene* genome. Scaffolds comprising each chromosome are indicated by alternating light and grey stripes. *Grs* without CNVs are indicated by open boxes and *Grs* with CNVs are indicated by closed boxes. *Grs* are classified as conserved if, in the *H. melpomene* reference genome, they have a one-to-one orthologous relationship with either a gene in *Danaus*, *Bombyx* or both (red dots, Figure 3). *Grs* are classified as non-conserved if they are duplicated in the *H. melpomene* reference genome or have no orthologue in either *Danaus*, *Bombyx* or both. Genes mapped to chromosomes but without precise locations are indicated by question marks. Scaffold arrangement is based on the published linkage map [13].

**Figure 8:**
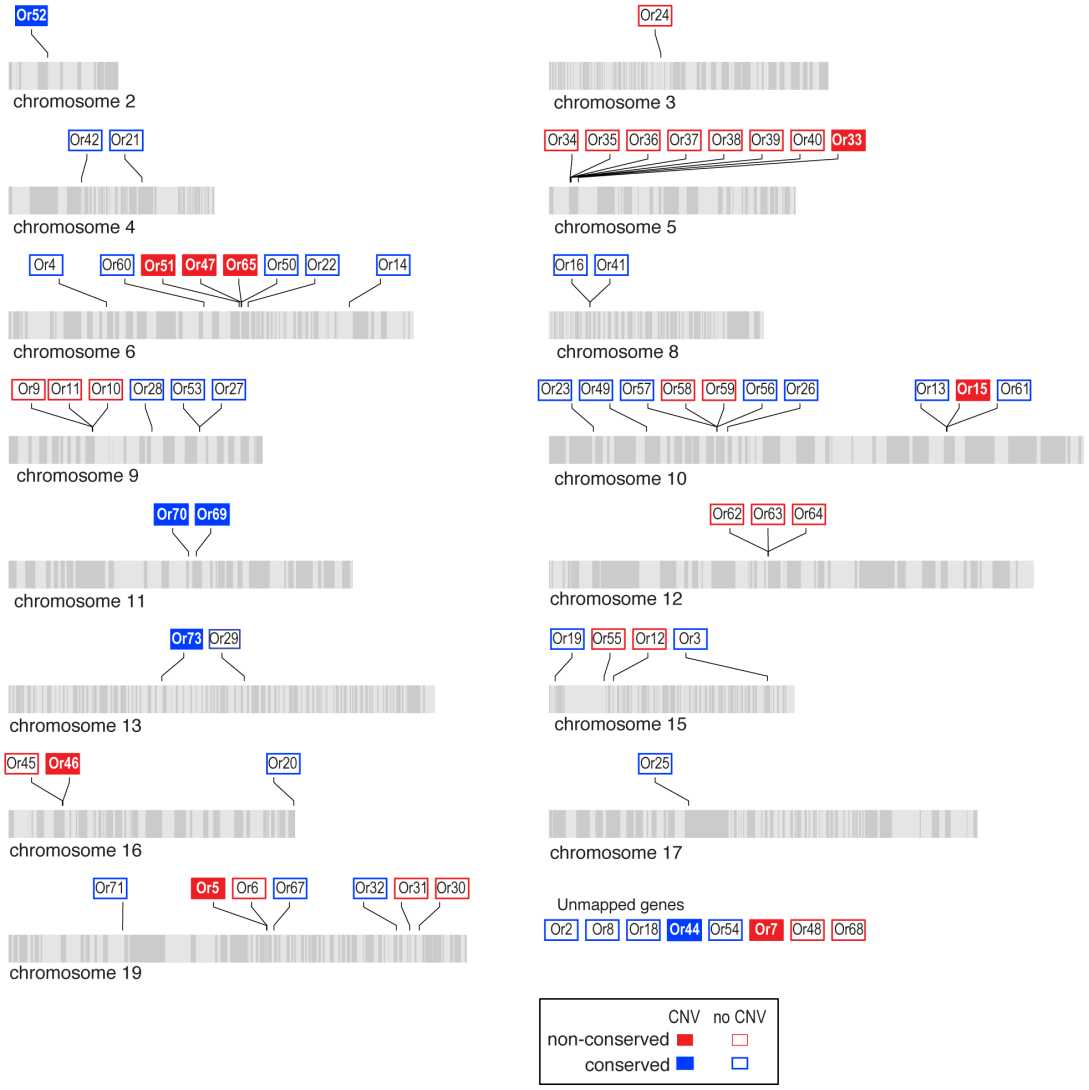
Copy-number variant (CNV) analysis of *Ors* in the *H.* *melpomene* genome. Scaffolds comprising each chromosome are indicated by alternating light and grey stripes. *Ors* without CNVs are indicated by open boxes and *Ors* with CNVs are indicated by closed boxes. The classification of *Ors* as being either conserved or non-conserved follows the same criteria as for the *Grs*. The eight genes for which the chromosome locality is not known are shown at the bottom.

We next tested whether the greater level of diversification of *Grs* as compared to *Ors* over long evolutionary timescales (compare Figure 3 and Figure 4), is similarly reflected in greater population level variation in *Gr* and *Or* duplicate genes. To test this hypothesis, we examined the incidence of CNVs among *Grs* and *Ors* that exist as single-copy genes in the reference *H. melpomene* genome with a one-to-one orthologous relationship with a gene in *Danaus*, *Bombyx* or both (conserved)(red dots, Figure 3 and 4), or as genes that are *Heliconius*-specific where no orthologue exists in either *Danaus* or *Bombyx* (non-conserved). We used whole genome resequence data (12 genomes) for three subspecies of *H. melpomene* (*H. melpomene amaryllis*, n = 4; *H. melpomene aglaope*, n = 4; and *H. melpomene rosina*, n = 4)(Figure 7, inset) and one sub-species of *H. cydno* (*H. cydno chioneus*, n = 4)(Table S4). We first mapped genomic resequence reads to the *H. melpomene melpomene* reference genome, and then searched for regions of abnormal coverage using CNVnator [56]. More than half of *Gr* loci showed presence of CNVs (37 out of 68 loci). However, there were noticeably fewer CNVs in *Gr* loci that evolve conservatively over the long-term, such as among the putative CO_2_ receptors, while there was an excess of CNVs in loci that show patterns of *Heliconius*-specific duplication (11.1% vs. 54.9%, respectively)(Fisher's Exact Test, two-tailed, *P* = 0.0004) (Table 1)(Figure 7). Intriguingly, many sugar receptor CNVs are sub-species specific; we observed fixed duplications relative to the reference genome in *H. melpomene aglaope* (*HmGr4, Gr5, Gr6, Gr8, Gr45, Gr52*) and *H. melpomene amaryllis* (*Gr4, Gr5, Gr6, Gr7, Gr8, Gr45, Gr52*), among genes that are found on different chromosomes (Table S5, Figure 7). Although the majority of CNVs are likely to be evolving neutrally, this raises the possibility of local adaptation within the species range around the detection of sugars. As expected given their long-term stability, *Ors* also show a lower incidence of CNVs (12 out of 67 loci), with no association between gene duplication and CNV incidence at least in *H. melpomene* (Table 1, Table S6). In *H. cydno*, a slight excess of *Or* CNVs was observed in loci that resulted in paralogous genes over longer evolutionary timescales (Fisher's Exact Test, two-tailed, *P* = 0.0475)(Table 1)(Figure 8).

**Table 1:** Relationship between evolutionarily-conserved genes and copy-number variation (CNV).

Species	Gene family	Gene classification	Number of genes with		*P* value§
			CNV	No-CNV	
*H. melpomene*	*Grs* †	*Heliconius*-specific	28	23	
		CO_2_ receptors+other conserved *Grs* *	1	8	
		Sugar receptors	8	0	0.0004
*H. cydno*	*Grs* †	*Heliconius*-specific	10	41	
		CO_2_ receptors + other conserved *Grs*	0	9	
		Sugar receptors	0	8	0.247
*H. melpomene*	*Ors* ‡	*Heliconius*-specific	7	24	
		Conserved *Ors*	5	29	0.527
*H. cydno*	*Ors* ‡	*Heliconius*-specific	6	25	
		Conserved *Ors*	1	33	0.0475

* Consists of single-copy genes in *H. melpomene*; in the monarch or *Bombyx* genomes, homologues are either single-copy or duplicate genes with bootstrap support ≥80%.

§ Fisher's exact test, two-tailed.

† Excludes 3 Grs where read-mapping of the reference genome reads back to the reference assembly indicated areas of poor assembly: *Gr37*, *Gr39* and *Gr49*.

‡ Excludes 3 Ors where read-mapping of the reference genome reads back to the reference assembly indicated areas of poor assembly: *Or20*, *Or24*, *Or43*, *Or50* and *Or74*.

We have not experimentally verified the incidence of copy number variation in any of these genomes, and some of the regions identified as CNVs are likely to be false positives. To investigate the rate of false positives, we analysed resequence data from the reference genome itself and discovered 3 *Gr* and 3 *Or* CNVs, suggesting a false positive rate of around 4%. (We therefore excluded these loci from our statistical tests.) However, the fact that broad patterns of observed CNVs are consistent with the evolutionary patterns at deeper levels supports our conclusion that CNV, in the absence of strong purifying selection, is an important driver of gene family diversification. These results also provide a novel line of evidence that the butterfly *Grs* have a higher rate of evolutionary turnover as compared to *Ors*.

### Sexually dimorphic gustatory sensilla in adult legs mirror *Gr* expression diversity

The life histories of adult male and female butterflies are similar with respect to the need to find food and potential mates except that adult females are under strong selection to identify suitable host plants for oviposition. To ascertain host-plant identity, female butterflies drum with their legs on the surface of leaves before laying eggs [10]. This behaviour presumably allows the female to taste oviposition stimulants. Consistent with this behaviour, adult nymphalid butterfly legs are known to contain gustatory sensilla [57], and it has been reported that while nymphalid butterfly females have clusters of gustatory sensilla on their foreleg foretarsi, males lack these entirely [10], [58]. Here we confirm this mostly anecdotal evidence for sexual dimorphism using scanning electron microscopy (SEM). The mid- and hindlegs of both male and female *H. melpomene* have similar numbers of individual gustatory sensilla along their entire lengths, but there is a striking difference in their abundance and distribution on the foretarsi of the female forelegs. Unlike males, females exhibit cuticular spines associated with gustatory (trichoid) sensillae (n∼80 sensilla/foretarsus for females; n = 0/foretarsus for males) (Figure 2A) [10].

**Figure 9:**
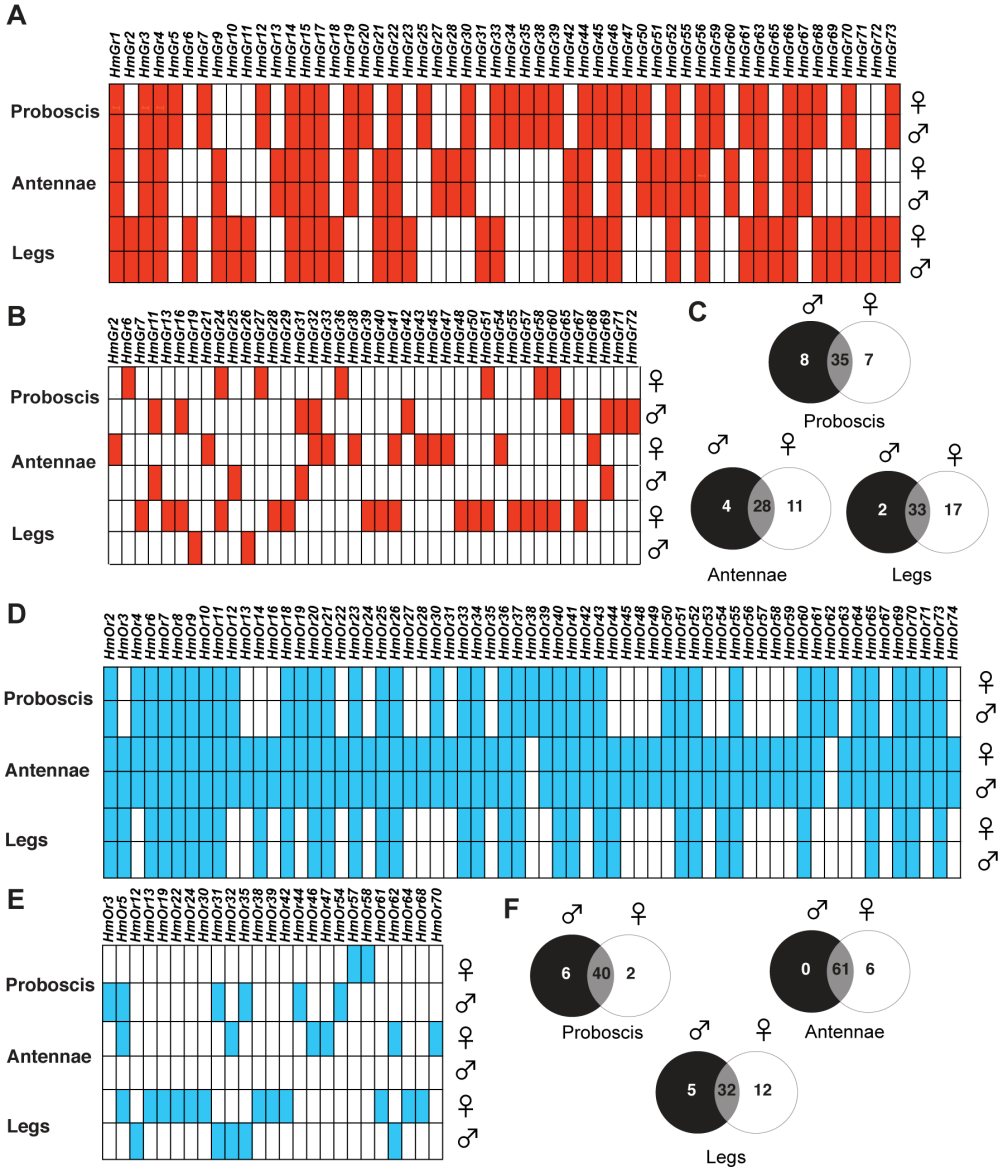
Comparison of *Gr* and *Or* expression in male and female adult *H.* *melpomene* chemosensory tissues. (A) The common set of *Grs* expressed in each tissue in both males and females. Red box indicates the presence of reads uniquely mapping to the coding region of each *Gr* gene model. To facilitate the visualization of tissue-specific expression found in both males and females, only *Grs* where both sexes show expression are indicated. Where only one sex or neither sex shows expression, the box is empty. (B) *Grs* showing sex-specific expression. To facilitate the visualization of sex-specific *Grs*, only *Grs* where one sex shows expression are indicated by a filled box. *Grs* which are expressed in both sexes or no sex are indicated by an empty box. (C) Venn diagram showing the number of uniquely expressed gustatory receptors in each transcriptome. (D) The common set of *Ors* expressed in each tissue in both males and females. Blue box indicates the presence of reads uniquely mapping to the coding region of each *Or* gene model. As above, only *Ors* where both sexes show expression are indicated. Where only one sex or neither sex show expression, the box is empty. (E) *Ors* showing sex-specific expression are indicated by a filled box. *Ors* which are expressed in both sexes or no sex are indicated by an empty box. (F) Venn diagram showing the number of uniquely expressed gustatory receptors in each transcriptome. The proboscis libraries also included both labial palps, the antennal libraries included both antennae, and the leg libraries included all six legs.

We therefore hypothesized that the repertoire of expressed *Gr* and *Or* genes in *H. melpomene* legs might be more diverse in females as compared to males. Furthermore, if female-specific genes are used for assessment of potential host plants, then fast-evolving insect-host interactions might produce rapid duplication of these genes over evolutionary timescales. Accordingly, we examined the expression profiles of *Grs* and *Ors* in adult *H. melpomene* by RNA-sequencing of libraries prepared from mRNAs expressed in adult antennae, labial palps and proboscis, and legs from one deeply-sequenced male and female each of *H. melpomene* (6 libraries total)(Table S7 and S8). The number of 100 bp reads per individual library ranged from 17.4 to 25.9 million for paired-end sequencing or 74.8–103.9 million for single-end sequencing (Table S8). To confirm these findings, we subsequently made 12 individual libraries from two more males and two more females (Table S7). As coverage was uneven across these libraries, we analysed them by merging biological replicates by sex and tissue type, and then downsampling so that an equal number of reads was analyzed for each treatment. The number of 100 bp reads analyzed for paired-end sequencing ranged from 19.4 to 49.6 million (Table S8). After downsampling, we examined the expression levels of the widely-expressed *elongation factor-1 alpha* gene in each of the libraries as a control, and found a comparable level of expression between sexes within each tissue type (Table S8). By careful visual examination of the uniquely-mapped reads to our 143 reference *Gr* and *Or* sequences, we found evidence of *Gr* and *Or* expression in all three adult tissue-types, with both tissue-specific and sex-specific differences as detailed below (Figure 9, Tables S9, S10, S11, S12, S13, S14). In total, we found evidence for expression of 67 of 73 *Grs* and 67 of 70 *Ors* identified in the *H. melpomene* reference genome.

Strikingly, the sexual dimorphism of gustatory sensilla we observed among the foreleg foretarsi is reflected in *Gr* gene expression patterns. A total of thirty-two *Grs* are expressed in both male and female *H. melpomene* leg transcriptomes including three CO_2_ receptors, *HmGr1-3*, four putative sugar receptors *HmGr4*, *Gr6*, *Gr45* and *Gr52* and a fructose receptor, *HmGr9* (Figure 9A, Table S9, Supplementary Text). Many Grs showed sex-specific expression, however, with many more *Grs* in female (n = 46) as compared to male leg transcriptomes (n = 33)(Figure 9B, C).

In total 15 of these *Grs* expressed in female legs, *HmGr10*, *Gr24*, *Gr26*, *Gr29*, *Gr40*, *Gr41*, *Gr48*, *Gr50*, *Gr51*, *Gr16*, *Gr55*, *Gr57*, *Gr58*, *Gr60* and *Gr67*, are the result of duplications since *Heliconius* and *Danaus* shared a common ancestor (Figure 3 small arrows, Figure 9B, Table S9). By contrast, only one of the three male-biased *Grs*, *HmGr19*, evolved as a result of recent duplication. There is an excess of *Heliconius*-specific *Grs* but not *Ors* (see below) that are expressed in female legs (Fisher's Exact Test, two-tailed, *p = 0.019*)(Table 2). Since male *H. melpomene* do not need to identify host-plants for oviposition, it seems likely that the 17 female-specific *Grs* in our leg transcriptomes are candidate receptors involved in mediating oviposition (Figure S1).

**Table 2:** An overabundance of *Grs* expressed in female legs are the result of *Heliconius*-specific duplication.

Gene Family	Gene duplication	Gene Expression		
		Female-specific	Both sexes	*P* value†
*Gr* ‡	*Heliconius*-specific	15	20	0.019
	Conserved*	1	13	
*Or* §	*Heliconius*-specific	6	12	0.483
	Conserved*	5	19	

* Consists of single-copy genes in *H. melpomene*; in the monarch or *Bombyx* genomes, homologues are either single-copy or duplicate genes with bootstrap support ≥80%.

† Fisher's exact test, two-tailed, d.f. = 1.

‡ Excludes *Gr39* because of poor coverage in the reference genome read-mapping.

§ Excludes *Or20* and *Or24* because of coverage in the reference genome.

### Female *Gr* expression is more diverse in antennae than male *Gr* expression

Besides using their antennae for olfaction, female nymphalid butterflies also taste a host plant by antennal tapping before oviposition. This tapping behaviour presumably allows the host plant chemicals to come into physical contact with gustatory sensilla on the antennae. We therefore examined whether there was any difference in the abundance of gustatory sensilla on the antennae of male and female *H. melpomene*. Using scanning electron microscopy, we found individual gustatory sensilla scattered along each antennae of both male and female *H. melpomene* but no obvious sexual dimorphism in their abundance or distribution (Figure 2B). We found 28 *Grs* expressed in both male and female *H. melpomene* antennae (Figure 9A, Table S10), including two sugar receptors, *HmGr4* and *HmGr52*, a putative fructose receptor *HmGr9* and two CO_2_ receptors, *HmGr1* and *Gr3*. Besides the sugar and CO_2_ receptors noted, other conserved genes that are expressed in both male and female antennae include *HmGr63*, a candidate Gr co-receptor (see Text S1), and *HmGr66*, a candidate bitter receptor.

We also found 11 *Grs* expressed in female *H. melpomene* antennae that did not appear to be expressed in male antennae. Two of these, *HmGr47* and *Gr68*, appeared in the top one-third of the most abundant female antennal *Grs* in terms of number of reads recovered from the individual butterfly transcriptome. In contrast, just four *Grs* were expressed in male antennae *HmGr11*, *Gr25*, *Gr31*, and *Gr69* but not female antennae (Figure 9B, C, Table S10). Six of the female-biased *Grs* and two of the male-biased *Grs* (*Gr31*, *Gr69*) expressed in antennae are the result of duplication events since *Heliconius* and *Danaus* shared a common ancestor.

### Candidate *Heliconius* gustatory receptors for nectar- and pollen-feeding

By contrast with the leg and antennal tissue, where more *Grs* are expressed in females compared to males, the labial palps and proboscis (Figure 1) transcriptomes contained the largest number of *Grs* (n = 35) expressed in both sexes (Figure 9A, C, Table S11). Five of the six candidate sugar receptors in the *H. melpomene* genome are expressed in both the male and the female transcriptomes along with two of the three conserved CO_2_ receptors, which may be used to assess floral quality [59] (Figure 3, Table S11). A majority (21 of 35) of *Heliconius Grs* expressed in both male and female labial palps and proboscis libraries have no existing ortholog in the silkmoth genome, apparently the result of gene loss in *B. mori* or gene duplication along the lineage leading to *Heliconius* (Figure 3). This may in part reflect the fact that adult silkmoths have lost the ability to feed. Interestingly, four *Grs* expressed in both male and female labial palps and proboscis transcriptomes could not be detected in male and female antennae and legs (*HmGr12*, *Gr20*, *Gr35*, and *Gr59*)(Figure 3, red arrows, Figure 9B). Some of these Grs might play a role in the pollen-feeding behaviour that is specific to *Heliconius*, and which involves preferences for particular species of flowers in the plant families Rubiaceae, Cucurbitaceae and Verbenaceae (see Discussion).

### Widespread expression of *Ors* in *H. melpomene* antennae, proboscis and labial palps and legs

In addition to the *Gr* gene expression described above, we examined *Or* expression in the three adult tissues. The expression of *Ors* in antennal tissue has been widely studied in a variety of insects including *Drosophila* and some Lepidoptera [50], [60]. As expected, we observed that *Or* gene expression was high in the antennae. Unexpectedly, *Or* expression was about as prevalent as *Gr* expression in the proboscis and labial palps and leg transcriptomes (Figure 9D, E, F). In total across all three tissues profiled, we found evidence for the expression of nearly all predicted *Or* genes (67 of 70 genes)(Table S12, S13, S14) in the *H. melpomene* reference genome [13].

## Discussion

**Figure 10:**
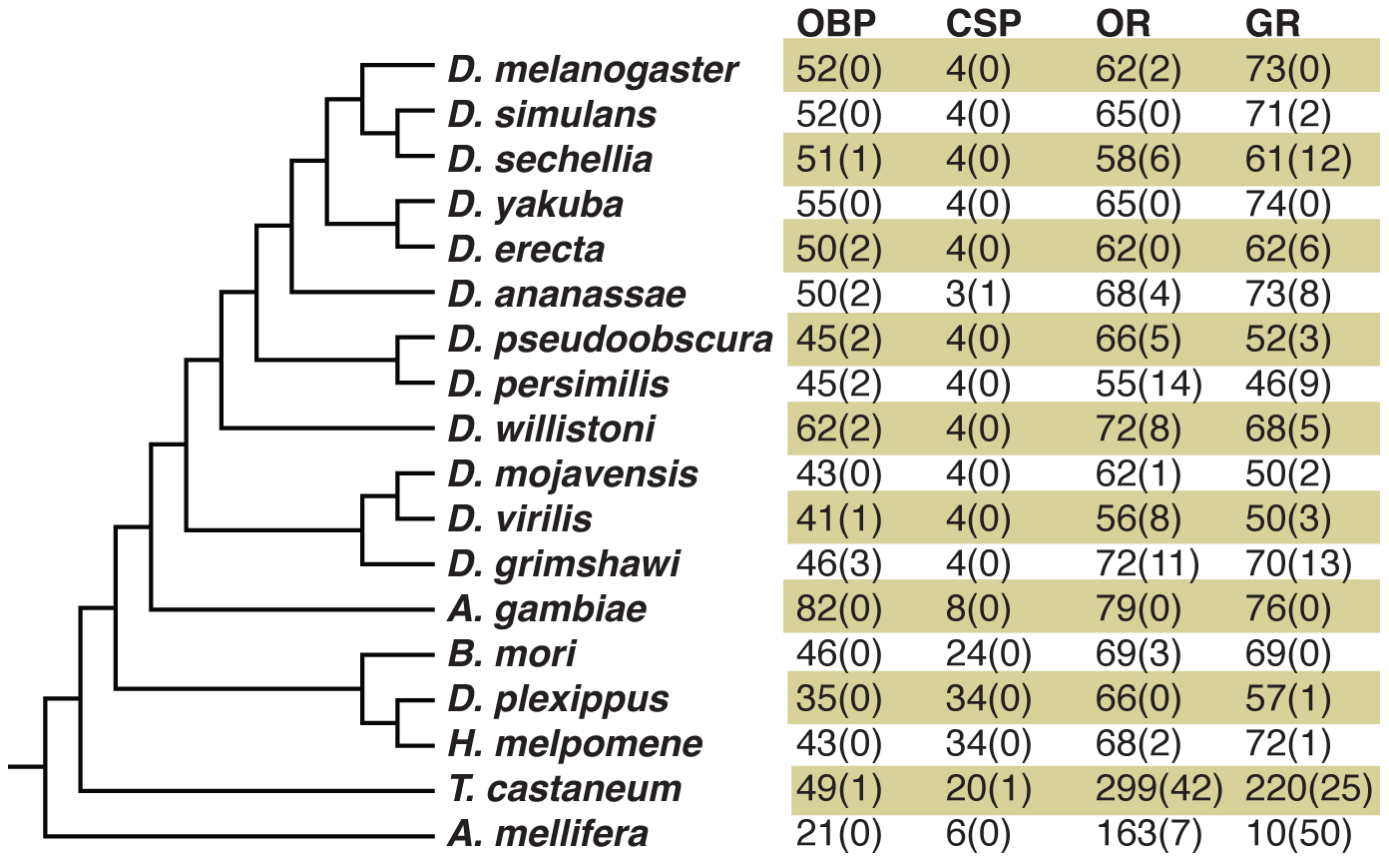
Insect chemosensory gene family repertoires. Numbers indicate intact genes and numbers in parentheses indicate pseudogenes. References are given in [13], [55], [94]. OBP = odorant binding protein; CSP = chemosensory protein; OR = olfactory receptor, GR = gustatory receptor.

Outside *Drosophila*, the study of sensory gene family evolution in insects has generally been limited to the comparison of a small number of phylogenetically distant reference genomes. Such studies have commonly involved a comparison of the size of gene families between taxa in order to document lineage-specific expansions (Figure 10), and the comparison of dN/dS ratios to identify branches subject to rapid evolution [61]. Here we have used a similar approach to annotate 73 *Grs* in the *Heliconius melpomene* reference genome. However, we have also demonstrated the power of next-generation sequencing to elucidate patterns of evolution and expression of these genes. These data have offered exciting new insights into a set of genes that show both rapid evolution and sex-specific expression patterns, suggesting that female oviposition behaviour drives the evolution of butterfly gustatory receptors.

Previous work in other insects indicates that *Grs* are an important target for gene duplication and loss between species. Most notably, *D. sechellia* and *D. erecta* are host specialists, on *Morinda citrifolia* and *Pandanus candelabrum* respectively, while *D. simulans* is a generalist fly exploiting a broad array of rotting fruit [46]. Host specialization in the former species is associated with an acceleration of gene loss and increased rates of amino acid evolution at receptors that remain intact. Here we have used whole-genome Illumina sequencing of single diploid individuals to similarly document patterns of gene gain and loss across *Heliconius*. This method yields highly fragmented genome assemblies, but such assemblies have proven very informative, most notably for studying the evolution of the clade of single-exon bitter receptor genes. We identified three gene duplication events along the lineage leading to *Heliconius*, followed by eight independent instances of clade-specific pseudogenizations or losses of different members of the intronless *Grs*, *Gr22-26* and *Gr53*, within *Heliconius* and one instance within *Danaus plexippus* (Figure 5 and Figure 6). In both *Heliconius* and *Drosophila* gene gain and loss appear to primarily affect *Grs* that are presumed to respond to bitter compounds (Figure 3). To verify whether this pattern holds within the genus *Heliconius* for the remaining gene family members with more complex intron-exon structure will require better genome assemblies for multiple *Heliconius* species (Table S2).

These patterns of rapid gene gain and loss are mirrored by within-population variation in copy number. From 16 resequenced genomes for *H. melpomene* and its sister species *H. cydno*, we have shown that CNVs occur more commonly among the *Grs* than the *Ors* (Figure 7, 8, Table 1). Within the *Grs*, the bitter receptors of *H. melpomene* represent a class of genes that are both highly prone to lineage-specific duplication and commonly subject to population-level copy number variation. These putative bitter receptor genes are also more likely to show female-specific expression, especially in the legs, which suggests a role in insect-host chemical interactions (Table 2, Figure 3, Figure S1).

In human genomes, a tendency for CNV-rich areas to display higher dN/dS ratios and yield paralogous genes has been noted [62], along with an enrichment of CNVs in genes involved in immune function and in the senses (specifically in *Ors* which are unrelated to the insect *Ors*) [63], [64]. It is also widely known that copy-number variation is an important source of disease-causing mutations in humans [64]. With the exception of insecticide resistance in insects [65], [66], the spectrum of naturally-occurring copy-number variants is only just starting to be explored in *Drosophila* [67], [68] and non-model systems. Our results demonstrate the great utility of high throughput sequencing to reveal the naturally-occurring spectrum of CNVs that underlie gene family expansions in non-model systems, in traits of ecological relevance.

*Heliconius* butterflies have complex relationships with their Passifloraceae host plants. Some species are host-specialist, feeding on only one or a few *Passiflora* species, others specialise on particular sub-genera within *Passiflora*, while others are generalists, albeit within this one host plant family (Figure 6) [53]. The Passifloraceae is extremely chemically diverse, most notably in their diversity of cyanogenic glycosides that protect the plant from herbivores. It seems likely that coevolution of the butterfly chemosensory and detoxification system on the one hand, with the plant biochemical defense on the other, has played an important role in the evolution of this chemical arsenal. In contrast to the research already carried out on the chemistry of the host plants [54], until recently almost nothing was known about the chemosensory system of *Heliconius* butterflies. All of these insect host-plant interactions are mediated primarily by adult female butterflies, which must correctly identify suitable host plants for oviposition [69], [70], or risk the survival of their offspring.

Expression data for *Grs* in the Lepidoptera have been limited until now–especially for adults–due to their low expression level. The largest previous study identified 14 *Grs* profiled in larval *B. mori* [32]. We have found evidence for adult expression for most (∼91%) of the 73 predicted *Gr* genes. This provides a marked contrast to the handful of gustatory receptors that have been identified from traditional expressed sequence tag (EST) projects in other Lepidoptera. Our methods may provide a greatly improved yield of expressed genes because we now have a set of well-annotated target *Gr* genes against which RNA-seq data can be mapped, together with a greater diversity of transcripts afforded by deep sequencing. Such methods have also permitted us to find widespread expression of their sister gene family, the *Ors*, in the adult chemosensory tissues examined (68 of 70 or 97% of predicted genes) (Figure 9).

Many of these *Gr* genes are likely to be involved in the detection of host plant attractants as well as toxic secondary metabolites and thus allow the discrimination of suitable hosts. Most notably, there were a large number of *Heliconius*-specific *Grs* with female-biased expression in both legs and antennae (Figure 9). As mentioned previously, these female-biased leg *Grs* (but not *Ors*) are also more likely to represent unique duplicates on the *Heliconius* lineage (Table 2). Female-biased *Or* expression, as quantified using RNA-seq data, has been reported for *Ors* expressed in the antennae of the adult mosquito, *Anopheles gambiae* [71]. Specifically, 22 *Ors* displayed enhanced expression in mosquito female antennae but not in male antennae. Since adult mosquito females but not males need to find hosts for a blood-meal, and adult butterfly females but not males need to find host plants for egg-laying, this suggests that host-seeking behaviour of female insects may be an important general driver of sensory gene evolution. Indirect evidence for the possible role of some of these Grs in *Heliconius* host plant detection comes from comparative studies of Grs mediating oviposition behaviour in swallowtail butterflies (Papilionidae). *Papilio xuthus* PxGr1 a member of the Gr subgroup that contains *D. melanogaster* Gr43a and HmGr9, has been characterized as a receptor for synephrine, which is an alkaloid found in citrus trees [52]. It is expressed in female *P. xuthus* tarsi and is necessary for the correct oviposition behavior of swallowtail butterflies [52]. Within the two clades most closely-related to *PxGr1*, are 9 butterfly-specific *Grs*: *HmGr10*, *Gr16*, *Gr55*, *Gr56* and *Gr57*, and the newly-described *DpGr16*, *Gr50*, *Gr52*, and *Gr54* (Figure 3). Four these *Grs*, *HmGr16*, *Gr55*, *Gr56* and *Gr57*, result from *Heliconius*-specific gene duplications (i.e., no *Danaus* or *Bombyx* homologs). *Grs55-57* are also in the top ten most highly expressed *Grs* in female legs. The identification of these sex-biased leg *Grs* has provided an important starting point for future ligand specificity studies combining heterologous expression, electrophysiology, RNAi [51], assays of the proboscis-extension reflex, and female oviposition behavior.

Lastly, the patterns of *Gr* gene expression among different tissues and sexes has permitted us to identify a number of *Grs* that are strong candidates for mediating the remarkable pollen feeding behaviour that is unique to *Heliconius*, among the butterflies. The *Heliconius* proboscis contains at least two types of gustatory sensilla, hair-like *sensilla chaetica*, and *sensilla styloconica* (Figure 1). Like other butterflies, *Heliconius* respond to varying amounts of sugars including sucrose present in floral nectar [72]. Unlike other moths and butterflies, *Heliconius* actively collect pollen with their proboscides, preferentially from *Psychotria* (Rubiaceae), *Psiguria/Gurania* (Cucurbitaceae) and *Lantana* (Verbenaceae) flowers [17], [18], [73]. Once a pollen load is collected (Figure 1D), the butterflies use a combination of mechanical shearing (coiling and uncoiling of the proboscis) and enzymatic activity (using proteases found in saliva) to release amino acids from the pollen [74]. The RNA-seq data we have collected for *H. melpomene* proboscis and labial palps should provide a useful resource for future studies examining the molecular basis of this unique digestive trait.

Pollen feeding in adult *Heliconius* has an important ecological function. Amino acids obtained from pollen are key resources used in male nuptial gifts and egg allocation [18], [75]–[77]. They also permit *Heliconius* adults to have exceptionally long lifespans. Pollen feeding behavior is not found outside the genus *Heliconius*, even in the sister genus *Eueides*, whose larvae share a preference for *Passiflora* host-plants with *Heliconius*. In the present study we have identified four *Heliconius*-specific *Grs* that are only expressed in the proboscis (*HmGr12*, *Gr20*, *Gr35* and *Gr59*) but not in antennae or legs (Figure 9B), suggesting a role for these genes in pollen-feeding behaviour.

Taken together, the whole-genome and whole-transcriptome data suggest that *Gr* genes in particular are highly evolutionarily labile both on short and long evolutionary timescales, and begin to offer an insight into the likely molecular basis for the rapid coevolution observed between these butterflies and their host plants. Understanding the remarkable diversity underlying this ecological interaction at a molecular level has remained a challenge (but see [32], [52], [78], [79]). Thanks to technological innovations in sequencing, the genetic basis of taste and olfaction involved in host-plant adaptation in *Heliconius* is beginning to be uncovered.

### Conclusions

We have shown that like the opsin visual receptors [80], the chemosensory superfamily composed of constituent *Gr* and *Or* families in Lepidoptera show rapid gene family evolution, with higher rates of copy-number variation and gene duplication among the *Grs* than the *Ors*, as well as gene losses in the *Grs*. In particular, there is a group of putative bitter receptors that show female-specific expression in the legs and that are especially prone to gene duplication, providing new material for sensory diversification in the insect-host plant arms race. We have also shown, for the first time, widespread expression of *Ors* in non-antennal tissues in a lepidopteran. With the most comprehensive data set on *Gr* and *Or* expression in butterflies to date we are one step closer to identifying the sensory and molecular genetic basis of the *Heliconius*-*Passiflora* co-evolutionary race that inspired Ehrlich and Raven in 1964.

## Materials and Methods

### Genome annotation

tBLASTn searches were conducted iteratively against the *H. melpomene melpomene* genome (version v1.1) and haplotype scaffolds [13] using *B. mori* [28], [47] and *D. plexippus Grs* [14] as input sequences. For these *in silico* gene predictions, intron-exon boundaries were identified by first translating the scaffold nucleotides in MEGA version 5 [81], searching for exons identified in the tBLASTn searches, then back translating to identify splice junctions. Intron sequences were then excised to verify that the remaining exonic sequences formed an in-frame coding sequence. Insect Grs are defined by a conserved C-terminal motif TYhhhhhQF, where ‘h’ is any hydrophobic amino acid [21]. We inspected our predicted protein sequences for this motif or variants thereof, specifically ‘S’, ‘M’ or ‘K’ instead of a ‘T’ or ‘L’, ‘T’ or ‘I’ instead of ‘F’. In the handful of cases where we were unable to find the last short exon that contains this motif, final assignment to the *Gr* gene family was based on using the predicted amino acid sequence as a search string for either tBLASTn or BLASTp against the nr/nt Genbank database. Gene annotations were submitted to the EnsemblMetazoa database http://metazoa.ensembl.org/Heliconius_melpomene/Info/Index as part of the *H. melpomene* v. 2 genome release (for GeneIDs see Table S1). Chromosomal assignments were based on published mapping of scaffolds in the *H. melpomene melpomene* reference genome [13].

Following amino acid alignment using ClustalW, preliminary phylogenetic trees were constructed in MEGA using neighbor-joining and pair-wise deletion to identify orthologous relationships with *B. mori* and *D. plexippus* Grs. Reciprocal tBLASTn searches against the *B. mori* and *D. plexippus* genomes as well as searches using the protein2genome module in EXONERATE [82] were then performed in order to search for ‘missing’ *Grs* in those genomes. Final phylogenetic analysis was performed using a maximum-likelihood (ML) algorithm and JTT model on an amino acid alignment that was inspected by eye and manually adjusted. These results were compared to a ML tree made from a Clustal-Omega alignment [83] and were found to be nearly identical. Once the initial *H. melpomene Gr* gene predictions were obtained, EXONERATE, Perl scripts and manual annotations in Apollo [84] were used to produce gff3 files for submission of the annotated *H. melpomene* genome scaffolds to EMBL-EBI.

### RNA-sequencing

Butterfly pupae of *H. melpomene rosina* were obtained from Suministros Entomológicos Costarricenses, S.A., Costa Rica. Adult males and females were sexed and frozen at −80°C. Total RNAs were extracted separately from antennae, proboscis together with labial palps, and all six legs of three males and three females of *H. melpomene* using Trizol (Life Technologies, Grand Island, NY). A NucleoSpin RNA II kit (Macherey-Nagel, Bethlehem, PA) was used to purify total RNAs. Each total RNA sample was purified through one NucleoSpin RNA II column. Purified total RNA samples were quantified using a Qubit 2.0 Fluorometer (Life Technologies, Grand Island, NY). The quality of the RNA samples was checked using an Agilent Bioanalyzer 2100 (Agilent Technologies, Santa Clara, CA). 0.3–4.0 µg of purified total RNAs were used to make cDNA libraries. A TruSeq RNA sample prep kit (Illumina, San Diego, CA) was used to prepare 18 individual cDNA libraries. After being normalized according to their concentrations, the enriched individual libraries were pooled and then run on a 2% agarose gel. cDNA products ranging from 280 to 340 bp with an average of 310 bp were cut out and purified using a Geneclean III kit (MP Biomedicals, Solon, OH) to facilitate post-sequencing assembly. After being re-purified using Agencourt AMPure XP magnetic beads (Beckman Coulter Genomics, Danvers, MA), the cDNA pool was quantified using the Qubit 2.0 Fluorometer, and quality control-checked using the Agilent Bioanalyzer 2100. The cDNA pools were then normalized to 10 nM and run as either two paired-end or three single-end 100 bp runs on a HiSeq 2000 (Illumina, San Diego, CA) by the UCI Genomics High-Throughput Facility.

### RNA-seq assembly and read mapping

mRNA sequences were demultiplexed, trimmed and sorted using Python and Perl scripts. A single *de novo* assembly of the combined libraries was performed using CLC Genomics Workbench 5 to check for missing exons in our gene models. The 73 corrected *Gr* gene models and 70 *Or* gene models were then used as an alignment reference to perform unique read mapping of each individual chemosensory transcriptome. To determine if an individual *Gr* or *Or* was expressed in a given tissue, each of the 1716 individual *Gr* and *Or* mapping alignments was inspected by eye for uniquely mapped reads, and any spuriously-mapped reads (i.e., reads <70 bp in length with indels or sequence mismatches at the ends) were discarded. As a control for potential differences in RNA preparation between samples, we also quantified the number of uniquely mapped fragments to the widely-expressed *elongation factor 1-alpha (EF1α)* gene transcript and calculated the Fragments Per Kilobase of transcript per Million mapped reads (FPKM) [85]. Illumina reads for each of the libraries were deposited as fastq files in the ArrayExpress archive under the accession number: E-TAB-1500 (Table S7).

### Scanning electron microscopy

One week old adult *H. melpomene rosina* butterflies were sexed, frozen at −80°C, then dissected and mounted for imaging on an FEI/Philips XL30 FEG scanning electron microscope at UCI's Materials Characterization User Facility. Forelegs, middle legs, hindlegs and antennae were examined for the presence of gustatory sensilla.

### Copy number variation analysis

We also examined resequenced genomes of twelve *H. melpomene* and four *H. cydno* individuals, including *H. melpomene aglaope*, *H. melpomene amaryllis* and *H. melpomene rosina* (Table S4), sequenced by The GenePool, University of Edinburgh, U.K. and the FAS Center, Harvard University, U.S.A., for evidence of copy-number variation (CNV) in the *Grs* and *Ors* using CNVnator [56]. These sequences were deposited in the European Nucleotide Archive (ENA) under accession number: ERP002440. The Illumina resequenced genomes were first mapped to the *H. melpomene* reference genome and the average read depth was calculated along a 100 bp sliding window. The output of CNVnator was parsed for candidate insertion and deletion variants, and those with estimated copy number of >2× were counted as potential duplications and <0.5× as potential deletions.

### Whole-genome sequence assembly

The GenePool, University of Edinburgh, and the Oxford Genomics Centre, University of Oxford, U.K., produced whole genome 100 bp sequences from *H. cydno*, *H. timareta*, *H. wallacei*, *H. doris*, *H. clysonymus*, *H. telesiphe*, *H. erato petiverana*, *H. sara* and *H. sapho* using the Illumina Pipeline v. 1.5–1.7 with insert sizes ranging from 300 to 400 bp. We deposited sequences for *H. sapho* and *H. sara* in the Sequence Read Archive (SRA) under accession number ERP002444. We performed *de novo* assembly of the short reads using Abyss v. 1.2 [86] implemented in parallel at the School of Life Sciences, University of Cambridge, U.K. Based on previous results [87], recommendations estimated by the software, and comparison of N50 values in preliminary experiments, we chose a k-mer size of 31, a minimum number of pairs required n = 5 and the minimum mean k-mer coverage of a unitig c = 2 (full command: abyss-pe n = 5 k = 31 c = 2 in = ‘for.fastq rev.fastq’). In all assemblies, at least 96% of reads mapped back to the contigs. We created BLAST databases of these whole genome sequence assembly contigs (Table S2) in Geneious Pro v. 5.5.6. The lack of introns in the putative bitter receptor genes *Gr22-26* and *Gr53* permitted us to easily retrieve them from these BLAST databases. To confirm the identity and improve the quality of the sequences found, we mapped the reads to the assembled exon sequences in CLC Genomics Workbench v. 5.5.1, using the following conservative settings to prevent mis-mapping of paralogous sequences: mismatch, insertion and deletion cost of 3; length fraction and similarity fraction of 0.9. We then inspected all read-mappings by eye. Because the intronless *Grs* are closely related, we aligned the translated nucleotide sequences in MEGA using the ClustalW algorithm, and also inspected the alignment by eye. For all intronless *Gr* sequences except for the pseudogenes, sequence length was highly conserved (i.e., there were few indels). To illustrate the high substitution rate of the retrieved pseudogene sequences, we selected the neighbor-joining method for tree reconstruction and performed 500 bootstrap replicates.

### Inferring gene duplications and losses

To infer the number of intronless *Gr* gene duplications and losses, we used the program Notung v. 2.6 [88], [89], which reconciles gene trees onto the species tree. The gene tree was made by a maximum likelihood analysis of 1074 nucleotide sites, aligned by Clustal-Omega, and 500 bootstrap replications. The species tree was derived from a phylogeny based on independent nuclear and mitochondrial DNA sequences [90].

### RT-PCR

We verified the presence of *HmGr22* in several adult tissues using reverse-transcriptase PCR and primers for *HmGr22* (5′-CCATAATTTTGTCATCCT-3′ and 5′-GATTTCGAAATAAGGTCTGT-3′) and *EF1*alpha (5′-CGTTTCGAGGAAATCAAGAAGG-3′ and 5′-GACATCTTGTAAGGGAAGACGCAG 3′). RNA was extracted from fresh frozen specimens using Trizol and purified using the Nucleospin RNA II kit, which contains a DNAase-treatment step. RNA concentration was diluted to 12.5 µg/ml. Each 25 µl reaction had 2.5 µl 10× BD Advantage 2 PCR buffer, 2.5 µl dNTPs (2 mM), 0.5 µl (100 µM) forward and 0.5 µl reverse primer, 0.5 µl (1∶20 diluted) Stratagene Affinity Script Reverse Transcriptase, 0.5 µl 50× Advantage 2 Polymerase Mix, 17 µl H_2_O and 1 µl RNA. The PCR reaction consisted of 38 cycles of 95°C for 30 s, 55°C for 30 s, and 68°C for 55 s. The identity of the RT-PCR products was confirmed by Sanger sequencing.

## Supporting Information

Figure S1 “For Bitter or Worse: A Tale of Sexual Dimorphism and Good Taste”, an original cartoon by author and illustrator of science-oriented comics, Jay S. Hosler.
(PDF)

Table S1 *Heliconius melpomene* genome gustatory receptor annotations. Gene name, EnsemblMetazoa GeneID, amino acid sequence, nucleotide sequence, number of exons, top BLAST hit.
(XLS)

Table S2 Whole genome Illumina sequencing *de novo* assembly statistics.
(DOC)

Table S3 Intronless gustatory receptor genes retrieved from whole-genome Illumina assemblies.
(DOC)

Table S4 CNV sample data and whole-genome resequencing statistics.
(DOC)

Table S5 CNVs in *H. melpomene* and *H. cydno* gustatory receptors.
(XLS)

Table S6 CNVs in *H. melpomene* and *H. cydno* olfactory receptors.
(XLS)

Table S7 List of specimens and localities used in RNA-seq.
(DOC)

Table S8 Number of 100 bp Illumina reads sequenced per RNA-seq library.
(DOC)

Table S9 Gustatory receptor mRNAs expressed in adult *H. melpomene* legs.
(DOC)

Table S10 Gustatory receptor mRNAs expressed in adult *H. melpomene* antennae.
(DOC)

Table S11 Gustatory receptor mRNAs expressed in adult *H. melpomene* labial palps and proboscis.
(DOC)

Table S12 Olfactory receptor mRNAs expressed in adult *H. melpomene* antennae.
(DOC)

Table S13 Olfactory receptor mRNAs expressed in adult *H. melpomene* legs.
(DOC)

Table S14 Olfactory receptor mRNAs expressed in adult *H. melpomene* proboscis and labial palps.
(DOC)

Text S1 Identification of *H. melpomene* homologs of all described insect Gr subfamilies.
(DOC)
