# Supplementary figures and images for: Female Behaviour Drives Expression and Evolution of Gustatory Receptors in Butterflies
Source: PLoS Genet. 2013 Jul 11;9(7):e1003620. doi: 10.1371/journal.pgen.1003620 (PMC3732137; doi:10.1371/journal.pgen.1003620)

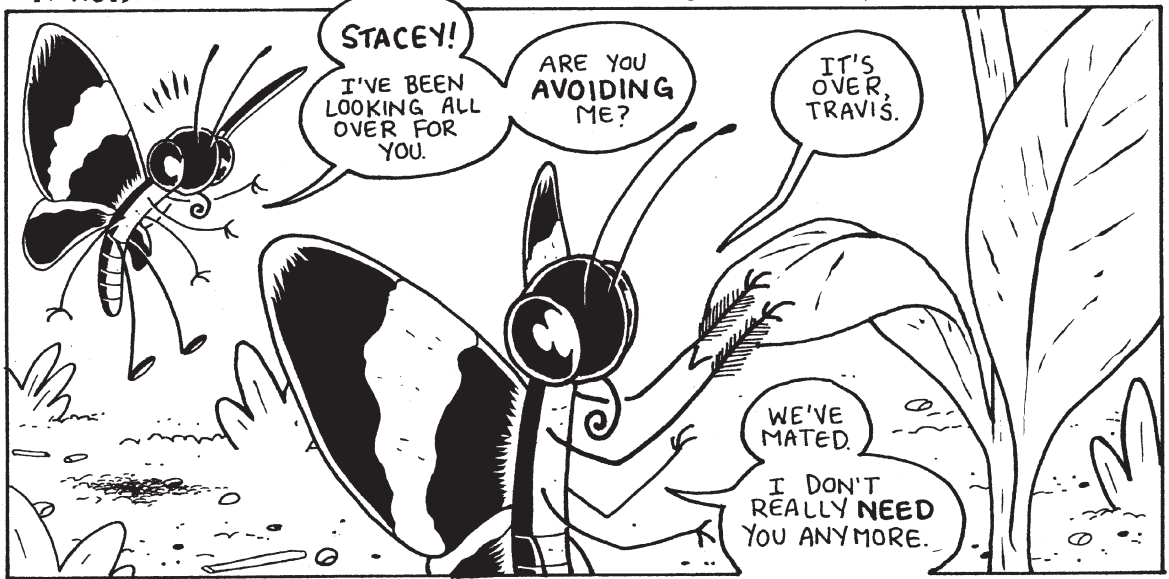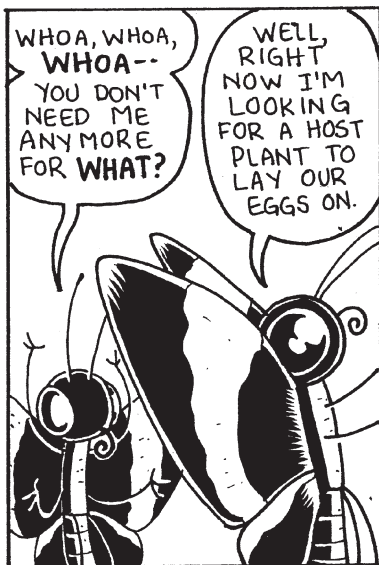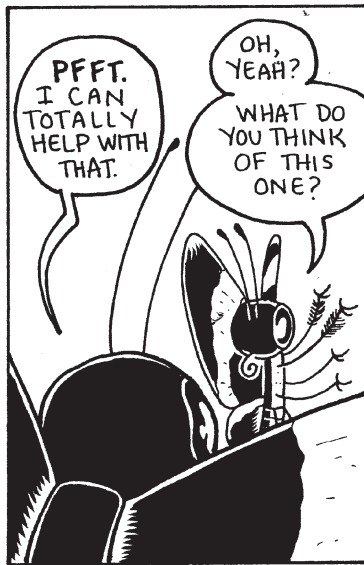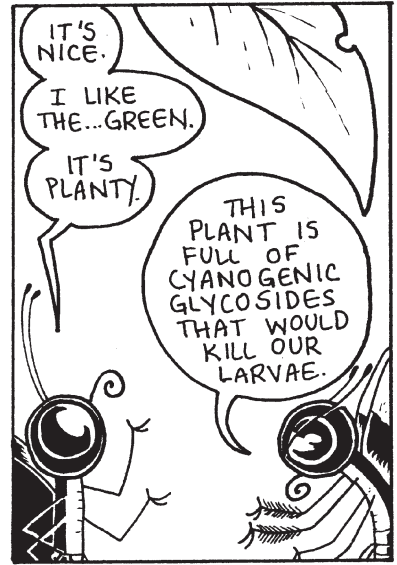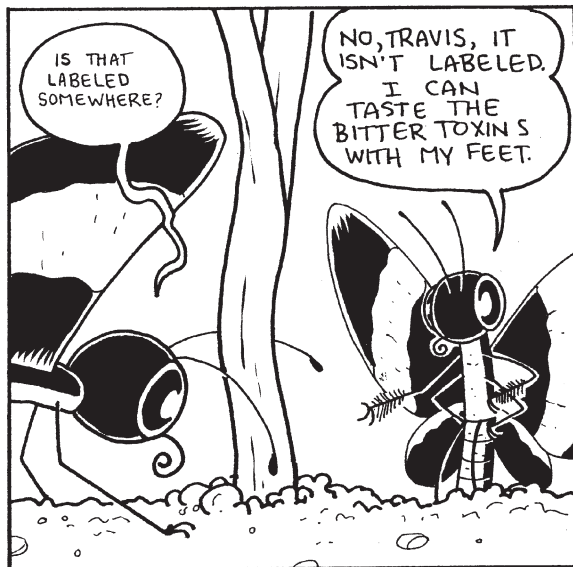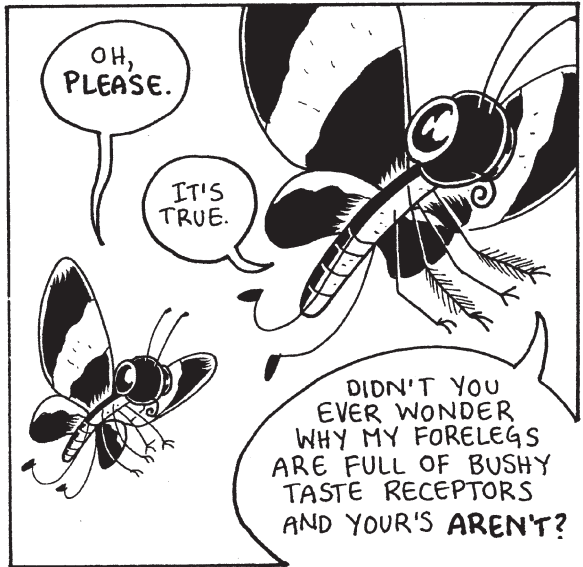

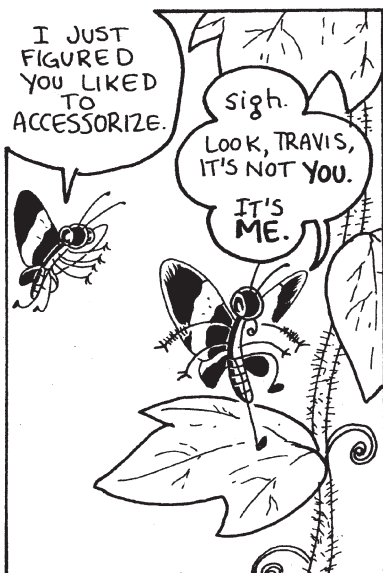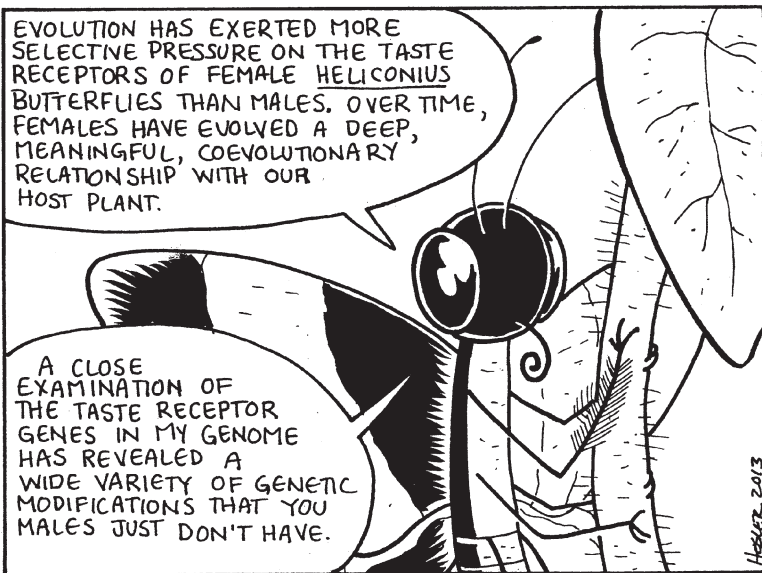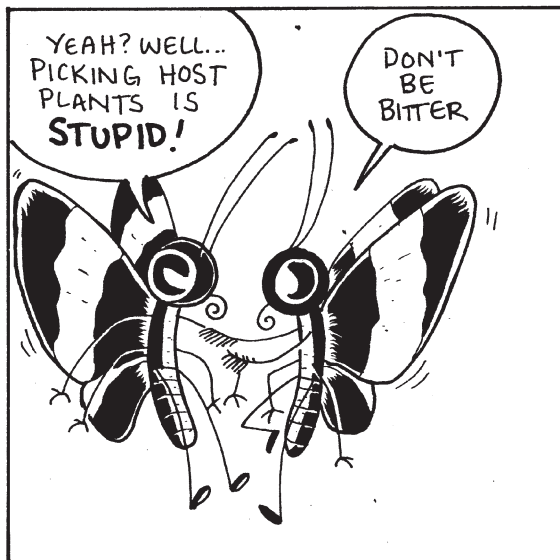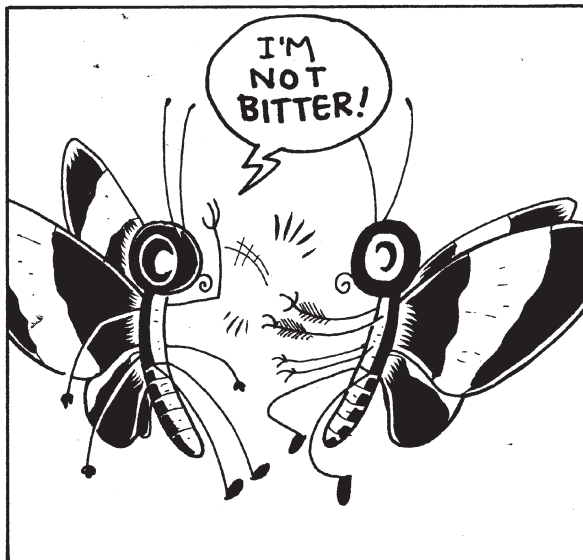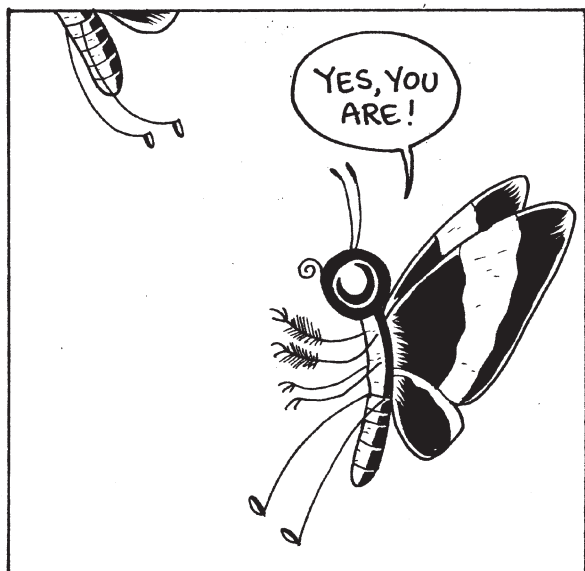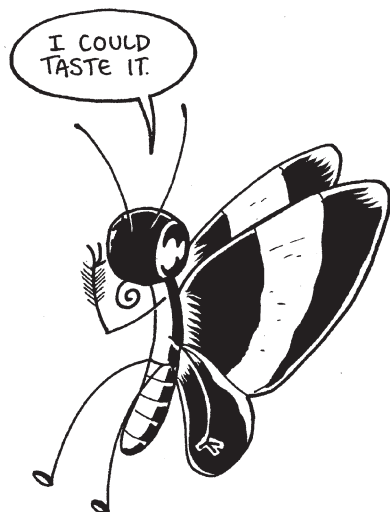

Supplement: Figure S1 — “For Bitter or Worse: A Tale of Sexual Dimorphism and Good Taste”, an original cartoon by author and illustrator of science-oriented comics, Jay S. Hosler. (PDF) [file pgen.1003620.s001.pdf]
