# Supplementary material for: Female Behaviour Drives Expression and Evolution of Gustatory Receptors in Butterflies
Source: PLoS Genet. 2013 Jul 11;9(7):e1003620. doi: 10.1371/journal.pgen.1003620 (PMC3732137; doi:10.1371/journal.pgen.1003620)
Supplement: Table S2 — Whole genome Illumina sequencing de novo assembly statistics. (DOC) [file pgen.1003620.s003.doc]

**Table S2. Whole genome Illumina sequencing *de novo*** assembly statistics.

| **Species** | **Individual** | **N reads** | **N:200** | **N50** | **Max length** | **Total Length** |
| --- | --- | --- | --- | --- | --- | --- |
| *H. cydno* | 564 | 121527124 | 441409 | 552 | 14116 | 214500000 |
| *H. clysonymus* | 40-9211 | 107073542 | 263462 | 1514 | 25811 | 274800000 |
| *H. erato* | 618 | 144079976 | 538193 | 708 | 19925 | 314400000 |
| *H. hecuba* | 36-8550 | 59631550 | 236780 | 1148 | 17102 | 202500000 |
| *H. telesiphe* | 37-16722 | 74889366 | 295734 | 940 | 14653 | 213800000 |
| *H. timareta* | 1-9178 | 121142840 | 240521 | 1348 | 15692 | 247100000 |
| *H. wallacei* | 10-8687 | 55005070 | 291516 | 1391 | 17547 | 293100000 |
| *H. doris* | 9-8684 | 85302970 | 250595 | 1060 | 12886 | 195200000 |
| *H. sara* | 8862 | 69531326 | 260389 | 1612 | 20437 | 303400000 |
| *H. sapho* | 260 | 90611610 | 219884 | 1921 | 21200 | 287100000 |

N:200 = total number of contigs > 200 bp

N50 = the length N for which half of all bases in the sequences are in a sequence of length L < N
