# Supplementary material for: Female Behaviour Drives Expression and Evolution of Gustatory Receptors in Butterflies
Source: PLoS Genet. 2013 Jul 11;9(7):e1003620. doi: 10.1371/journal.pgen.1003620 (PMC3732137; doi:10.1371/journal.pgen.1003620)
Supplement: Table S3 — Intronless gustatory receptor genes retrieved from whole-genome Illumina assemblies. (DOC) [file pgen.1003620.s004.doc]

**Table S3. Intronless gustatory receptor genes retrieved from whole-genome Illumina assemblies.**

| **Species** | **Locality** | **Gene Name** | **GenBank**  **Accession** | **Comments** |
| --- | --- | --- | --- | --- |
| *H. clysonymus* | El Topo, Tungurahua, Ecuador | *HclyGr23* | KC313949 | complete |
|  |  | *HclyGr24* | KC313950 | complete |
|  |  | *HclyGr25* | KC313951 | pseudogene |
|  |  | *HclyGr26* | KC313952 | complete |
|  |  | *HclyGr53* | KC313953 | complete |
| *H. cydno chioneus* | Pipe Line Road, Colon, Panama | *HcydGr22* | KC313959 | complete |
|  |  | *HcydGr23* | KC313954 | complete |
|  |  | *HcydGr24* | KC313955 | complete |
|  |  | *HcydGr25* | KC313956 | complete |
|  |  | *HcydGr26* | KC313957 | complete |
|  |  | *HcydGr53* | KC313958 | complete |
| *H. doris* | Pongo-Baranquita Rd, San Martin, Peru | *HdorGr22* | KC313960 | complete |
|  |  | *HdorGr24* | KC313961 | pseudogene |
|  |  | *HdorGr25* | KC313962 | complete |
|  |  | *HdorGr26* | KC313963 | complete |
| *H. erato petiverana* | Pipe Line Road, Colon, Panama | *HeraGr23* | KC313964 | complete |
|  |  | *HeraGr23* | KC313965 | complete |
|  |  | *HeraGr24* | KC313966 | complete |
|  |  | *HeraGr26* | KC313967 | complete |
|  |  | *HeraGr53* | KC313968 | complete |
| *H. hecuba* | Ecuador | *HhcbGr22* | KC313969 | complete |
|  |  | *HhcbGr24* | KC313970 | complete |
|  |  | *HhcbGr25* | KC313971 | complete |
|  |  | *HhcbGr26* | KC313972 | complete |
|  |  | *HhcbGr53* | KC313973 | complete |
| *H. melpomene rosina* |  | *HmelGr22* | KC313974 | complete |
| *H. sapho* | Mariquita Reserve, Tolima, Colombia | *HsapGr23* | KC313975 | complete |
|  |  | *HsapGr24* | KC313976 | complete |
|  |  | *HsapGr26* | KC313977 | complete |
|  |  | *HsapGr53* | KC313978 | complete |
| *H. sara* | Cana, Darien, Panama | *HsarGr23* | KC313979 | complete |
|  |  | *HsarGr24* | KC313980 | complete |
|  |  | *HsarGr26* | KC313981 | complete |
|  |  | *HsarGr53* | KC313982 | complete |
| *H. telesiphe* | El Topo, Tungurahua, Ecuador | *HtelGr23* | KC313983 | 20 aas missing |
|  |  | *HtelGr24* | KC313984 | 17 aas missing |
|  |  | *HtelGr25* | KC313985 | pseudogene |
|  |  | *HtelGr26* | KC313986 | complete |
|  |  | *HtelGr53* | KC313987 | 35 aas missing |
| *H. timareta* | El Topo, Tungurahua, Ecuador | *HtimGr22* | KC313988 | complete |
|  |  | *HtimGr23* | KC313989 | complete |
|  |  | *HtimGr24* | KC313990 | 7 aas missing |
|  |  | *HtimGr25* | KC313991 | complete |
|  |  | *HtimGr26* | KC313992 | 3 aas missing |
|  |  | *HtimGr53* | KC313993 | complete |
| *H. wallacei* | Pongo-Baranquita Rd, San Martin, Peru | *HwalGr22* | KC313994 | complete |
|  |  | *HwalGr25* | KC313995 | 13 aas missing |
|  |  | *HwalGr26* | KC313996 | complete |
|  |  | *HwalGr53* | KC313997 | complete |
