# Supplementary material for: Female Behaviour Drives Expression and Evolution of Gustatory Receptors in Butterflies
Source: PLoS Genet. 2013 Jul 11;9(7):e1003620. doi: 10.1371/journal.pgen.1003620 (PMC3732137; doi:10.1371/journal.pgen.1003620)
Supplement: Table S4 — CNV sample data and whole-genome resequencing statistics. (DOC) [file pgen.1003620.s005.doc]

**Table S4. CNV sample data and whole-genome resequencing statistics**

| **ID** | **taxon** | **sex** | **country** | **latitude** | **longitude** | **mean**  **sequence**  **depth** |
| --- | --- | --- | --- | --- | --- | --- |
| 531^1^ | *H. melpomene rosina* | M | Panama | 9.1206 N | 79.6969 W | 26.9 |
| 533^1^ | *H. melpomene rosina* | M | Panama | 9.1206 N | 79.6969 W | 26.7 |
| 546^1^ | *H. melpomene rosina* | M | Panama | 9.1206 N | 79.6969 W | 26.5 |
| 2071^1^ | *H. melpomene rosina* | M | Panama | 9.1206 N | 79.6969 W | 36.7 |
| 1^1^ | *H. melpomene melpomene* | ? | Panama | 8.6136 N | 78.1398 W | 23.0 |
| 11-48^2^ | *H. melpomene amaryllis* | M | Peru | 6.0960 S | 76.9774 W | 55.6 |
| 11-160^2^ | *H. melpomene amaryllis* | F | Peru | 6.4685 S | 76.3533 W | 44.0 |
| 09-216^2^ | *H. melpomene amaryllis* | M | Peru | 5.6756 S | 77.6747 W | 32.6 |
| 11-293^2^ | *H. melpomene amaryllis* | F | Peru | 6.4703 S | 76.3473 W | 53.3 |
| 09-108^2^ | *H. melpomene aglaope* | M | Peru | 5.9103 S | 762258 W | 36.6 |
| 09-112^2^ | *H. melpomene aglaope* | M | Peru | 5.9103 S | 762258 W | 38.9 |
| 11-569^2^ | *H. melpomene aglaope* | M | Peru | 5.9458 S | 76.2453 W | 44.4 |
| 11-572^2^ | *H. melpomene aglaope* | M | Peru | 5.9458 S | 76.2466 W | 37.4 |
| 553^1^ | *H. cydno chioneus* | M | Panama | 9.1714 N | 79.7573 W | 35.8 |
| 560^1^ | *H. cydno chioneus* | M | Panama | 9.1714 N | 79.7573 W | 35.3 |
| 564^1^ | *H. cydno chioneus* | M | Panama | 9.1714 N | 79.7573 W | 39.2 |
| 565^1^ | *H. cydno chioneus* | M | Panama | 9.1714 N | 79.7573 W | 46.0 |

^1^ GenePool: The GenePool, University of Edinburgh

^2^ FAS: FAS Center for Systems Biology, Harvard University

Quality-filtered reads were submitted to the European Nucleotide Archive, accession number: ERP002440.
