# Supplementary material for: Female Behaviour Drives Expression and Evolution of Gustatory Receptors in Butterflies
Source: PLoS Genet. 2013 Jul 11;9(7):e1003620. doi: 10.1371/journal.pgen.1003620 (PMC3732137; doi:10.1371/journal.pgen.1003620)
Supplement: Table S7 — List of specimens and localities used in RNA-seq. (DOC) [file pgen.1003620.s008.doc]

**Table S7.** List of specimens and localities used in RNA-seq.

| **Species** | **Locality** | **ID No.** | **Sex** | **Tissue type** | **Library type** | **Illumina**  **Sequencing** | **ENA Run Accession Number** |
| --- | --- | --- | --- | --- | --- | --- | --- |
| *H. melpomene rosina* | Costa Rica | 347 | F | Antennae | RNA-seq | 100 bp PE | ERR232462 |
| *H. melpomene rosina* | Costa Rica | 347 | F | Proboscis + palps | RNA-seq | 100 bp PE | ERR232452 |
| *H. melpomene rosina* | Costa Rica | 347 | F | Legs | RNA-seq | 100 bp PE | ERR232444 |
| *H. melpomene rosina* | Costa Rica | 347 | F | Antennae | RNA-seq | 100 bp SE | ERR232454 |
| *H. melpomene rosina* | Costa Rica | 347 | F | Proboscis + palps | RNA-seq | 100 bp SE | ERR232461 |
| *H. melpomene rosina* | Costa Rica | 347 | F | Legs | RNA-seq | 100 bp SE | ERR232459 |
| *H. melpomene rosina* | Costa Rica | 502 | F | Antennae | RNA-seq | 100 bp PE | ERR232446 |
| *H. melpomene rosina* | Costa Rica | 502 | F | Proboscis + palps | RNA-seq | 100 bp PE | ERR232457 |
| *H. melpomene rosina* | Costa Rica | 502 | F | Legs | RNA-seq | 100 bp PE | ERR232463 |
| *H. melpomene rosina* | Costa Rica | 503 | F | Antennae | RNA-seq | 100 bp PE | ERR232458 |
| *H. melpomene rosina* | Costa Rica | 503 | F | Proboscis + palps | RNA-seq | 100 bp PE | ERR232445 |
| *H. melpomene rosina* | Costa Rica | 503 | F | Legs | RNA-seq | 100 bp PE | ERR232456 |
| *H. melpomene rosina* | Costa Rica | 406 | M | Antennae | RNA-seq | 100 bp PE | ERR232465 |
| *H. melpomene rosina* | Costa Rica | 406 | M | Proboscis + palps | RNA-seq | 100 bp PE | ERR232450 |
| *H. melpomene rosina* | Costa Rica | 406 | M | Legs | RNA-seq | 100 bp PE | ERR232455 |
| *H. melpomene rosina* | Costa Rica | 406 | M | Antennae | RNA-seq | 100 bp SE | ERR232464 |
| *H. melpomene rosina* | Costa Rica | 406 | M | Proboscis + palps | RNA-seq | 100 bp SE | ERR232453 |
| *H. melpomene rosina* | Costa Rica | 406 | M | Legs | RNA-seq | 100 bp SE | ERR232443 |
| *H. melpomene rosina* | Costa Rica | 500 | M | Antennae | RNA-seq | 100 bp PE | ERR232451 |
| *H. melpomene rosina* | Costa Rica | 500 | M | Proboscis + palps | RNA-seq | 100 bp PE | ERR232447 |
| *H. melpomene rosina* | Costa Rica | 500 | M | Legs | RNA-seq | 100 bp PE | ERR232460 |
| *H. melpomene rosina* | Costa Rica | 501 | M | Antennae | RNA-seq | 100 bp PE | ERR232466 |
| *H. melpomene rosina* | Costa Rica | 501 | M | Proboscis + palps | RNA-seq | 100 bp PE | ERR232448 |
| *H. melpomene rosina* | Costa Rica | 501 | M | Legs | RNA-seq | 100 bp PE | ERR232449 |

These fastq files may also be retrieved using ArrayExpress accession number: E-MTAB-1500.
