# Supplementary material for: Female Behaviour Drives Expression and Evolution of Gustatory Receptors in Butterflies
Source: PLoS Genet. 2013 Jul 11;9(7):e1003620. doi: 10.1371/journal.pgen.1003620 (PMC3732137; doi:10.1371/journal.pgen.1003620)
Supplement: Table S8 — Number of 100 bp Illumina reads sequenced per RNA-seq library. (DOC) [file pgen.1003620.s009.doc]

**Table S8. Number of 100 bp Illumina reads sequenced per RNA-seq library in tissues of three male and three female *H. melpomene*.**

|  | ***H. melpomene*** | | | | | |
| --- | --- | --- | --- | --- | --- | --- |
|  | **Male 1** | | | **Female 1** | | |
|  | **Paired-end** | ***EF1*-α**  **FPKM^†^** | **Single-end** | **Paired-end** | ***EF1*-α**  **FPKM^†^** | **Single-end** |
| Legs | 8,774,889 | 4555.3 | 103,910,745 | 8,727,151 | 1726.8 | 98,086,547 |
| Antennae | 12,227,115 | 2228.5 | 74,819,205 | 12,258,206 | 2297.8 | 101,614,940 |
| Proboscis | 12,777,885 | 2127.5 | 98,509,997 | 12,262,639 | 1781.8 | 94,250,608 |
|  | **Male 2 & 3^§^** | | | **Female 2 & 3^§^** | | |
|  | **Paired-end** | ***EF1*-α**  **FPKM** | **Single-end** | **Paired-end** | ***EF1*-α**  **FPKM** | **Single-end** |
| Legs | 9,732,727 | 2660.6 | N/A | 9,732,727 | 2881.2 | N/A |
| Antennae | 24,830,059 | 4379.6 | N/A | 24,830,059 | 4128.8 | N/A |
| Proboscis | 11,635,958 | 4403.0 | N/A | 11,635,958 | 3897.8 | N/A |

**^†^**FPKM=fragments per kilobase of *EF1***−-**α sequence per million fragments mapped against the *H. melpomene* reference genome.

**^§^**Reads from individual libraries of biological replicates 2 and 3 were combined and an equivalent number of paired-end reads was analyzed from the combined libraries.
