# Supplementary material for: Female Behaviour Drives Expression and Evolution of Gustatory Receptors in Butterflies
Source: PLoS Genet. 2013 Jul 11;9(7):e1003620. doi: 10.1371/journal.pgen.1003620 (PMC3732137; doi:10.1371/journal.pgen.1003620)
Supplement: Table S9 — Gustatory receptor mRNAs expressed in adult H. melpomene legs. (DOC) [file pgen.1003620.s010.doc]

**Table S9. Names of gustatory receptor mRNAs expressed in adult *H. melpomene*** legs.

| **Tissue** |  | **Sex** |  |  | **Lineage** | **Putative Function** |
| --- | --- | --- | --- | --- | --- | --- |
|  | Male 1 | Female 1 | Male 2 & 3 | Female 2 & 3 |  |  |
| Both sexes | *HmGr1* | *HmGr1* | *HmGr1* | *HmGr1* | *B, D, H* | CO_2_ |
|  | *HmGr2* |  |  | *HmGr2* | *B, D, H* | CO_2_ |
|  | *HmGr3* | *HmGr3* |  |  | *B, D, H* | CO_2_ |
|  | *HmGr4** | *HmGr4* |  |  | *B, D, H* | sugar |
|  | *HmGr6* | *HmGr6* |  |  | *B, D, H* | sugar |
|  | *HmGr9* | *HmGr9* |  |  | *B, D, H* | fructose |
|  | *HmGr10* | *HmGr10* |  | *HmGr10* | *H* | synephrine-related |
|  | *HmGr11** | *HmGr11* |  |  | *H* |  |
|  | *HmGr14* | *HmGr14* |  | *HmGr14* | *H* |  |
|  | *HmGr15* | *HmGr15* | *HmGr15* | *HmGr15* | *H* |  |
|  | *HmGr17* | *HmGr17* | *HmGr17* | *HmGr17* | *H* |  |
|  | *HmGr18* | *HmGr18* |  |  | *H* |  |
|  | *HmGr21** | *HmGr21** |  |  | *H* |  |
|  | *HmGr22* | *HmGr22* | *HmGr22* | *HmGr22* | *H* |  |
|  | *HmGr23* | *HmGr23* |  |  | *H* |  |
|  | *HmGr31* | *HmGr31* |  |  | *H* |  |
|  | *HmGr33* | *HmGr33* |  |  | *H* |  |
|  | *HmGr42* | *HmGr42* | *HmGr42* | *HmGr42* | *H* |  |
|  | *HmGr44* | *HmGr44* | *HmGr44* | *HmGr44* | *Duplicated in B, D* |  |
|  | *HmGr45* | *HmGr45* |  |  | *H, duplicated in D* | sugar |
|  | *HmGr46* | *HmGr46* |  | *HmGr46* | *B, H* |  |
|  | *HmGr52* | *HmGr52* |  |  | *H, duplicated in D* |  |
|  |  | *HmGr56* | *HmGr56* | *HmGr56* | *H* | synephrine-related |
|  | *HmGr61*^†^*/64/65* | *HmGr61*^†^*/64/65* | *HmGr61*^†^*/64/65* |  | *H* |  |
|  | *HmGr63* | *HmGr63* | *HmGr63* | *HmGr63* | *B, D, H* | co-receptor |
|  | *HmGr66** | *HmGr66** |  |  | *H, D, duplicated in B* |  |
|  | *HmGr68* | *HmGr68* | *HmGr68* |  | *B, D, H* |  |
|  | *HmGr69* | *HmGr69* | *HmGr69* |  | *H* |  |
|  | *HmGr70* | *HmGr70** |  | *HmGr70** | *H* |  |
|  | *HmGr71* | *HmGr71* |  | *HmGr71* | *H* |  |
|  | *HmGr72* | *HmGr72* |  | *HmGr72* | *H* |  |
|  |  | *HmGr73* | *HmGr73* | *HmGr73* | *H* |  |
|  |  | *HmGr26* | *HmGr26* |  | *H* |  |
|  |  |  |  |  |  |  |
| Male-specific | *HmGr19* |  | *HmGr19* |  | *H* | fructose |
|  |  |  | *HmGr26* |  | *H* |  |
|  |  |  |  |  |  |  |
| Female-specific |  | *HmGr7* |  |  | *B, D, H* | sugar |
|  |  | *HmGr13* |  |  | *H* |  |
|  |  | *HmGr16* |  | *HmGr16* | *H* | synephrine-related |
|  |  | *HmGr24* |  |  | *H* |  |
|  |  | *HmGr28* |  |  | *H* |  |
|  |  |  |  | *HmGr29* | *H* |  |
|  |  | *HmGr39* |  |  | *H* |  |
|  |  | *HmGr40* |  | *HmGr40* | *H* |  |
|  |  | *HmGr41* |  | *HmGr41* | *H* |  |
|  |  | *HmGr48* |  | *HmGr48* | *H* |  |
|  |  | *HmGr50* |  | *HmGr50* | *H* |  |
|  |  | *HmGr51* |  | *HmGr51* | *H* |  |
|  |  | *HmGr55* |  | *HmGr55* | *H* | synephrine-related |
|  |  | *HmGr57* |  | *HmGr57* | *H* | synephrine-related |
|  |  | *HmGr58* |  | *HmGr58* | *H* |  |
|  |  | *HmGr60* |  |  | *H* |  |
|  |  | *HmGr67* |  |  | *H* |  |

*B, D, H* indicates the orthologous gene is present in *Bombyx, Danaus* and *Heliconius* genomes, respectively. *H* indicates that the gene is present only in the *H. melpomene* genome.

*Amino acid substitutions in mapped reads compared to reference genome.

^†^Expressed pseudogene.
