# Supplementary material for: Female Behaviour Drives Expression and Evolution of Gustatory Receptors in Butterflies
Source: PLoS Genet. 2013 Jul 11;9(7):e1003620. doi: 10.1371/journal.pgen.1003620 (PMC3732137; doi:10.1371/journal.pgen.1003620)
Supplement: Table S10 — Gustatory receptor mRNAs expressed in adult H. melpomene antennae. (DOC) [file pgen.1003620.s011.doc]

**Table S10.** **Gustatory receptor mRNAs expressed in adult *H. melpomene* antennae.**

| **Tissue** | **Male 1** | **Female 1** | **Male 2 & 3** | **Female 2 & 3** | **Lineage** | **Putative**  **Function** |
| --- | --- | --- | --- | --- | --- | --- |
| Both sexes | *HmGr1* | *HmGr1* | *HmGr1* | *HmGr1* | *B, D, H* | CO_2_ |
|  | *HmGr3* | *HmGr3* | *HmGr3* | *HmGr3* | *B, D, H* | CO_2_ |
|  | *HmGr4* | *HmGr4** |  |  | *B, D, H* | sugar |
|  | *HmGr9* | *HmGr9* | *HmGr9* | *HmGr9* | *B, D, H* | fructose |
|  | *HmGr13* | *HmGr13* |  |  | *H, duplicated in D* |  |
|  | *HmGr14** | *HmGr14* |  |  | *H* |  |
|  | *HmGr15* | *HmGr15* | *HmGr15* | *HmGr15* | *H* |  |
|  | *HmGr17* | *HmGr17* | *HmGr17* | *HmGr17* | *H* |  |
|  | *HmGr19* | *HmGr19* |  |  | *H* |  |
|  | *HmGr21* | *HmGr21* |  |  | *H* |  |
|  | *HmGr22* | *HmGr22* | *HmGr22* | *HmGr22* | *H* |  |
|  | *HmGr27* | *HmGr27* |  |  | *H* |  |
|  | *HmGr28* | *HmGr28* | *HmGr28* | *HmGr28* | *H* |  |
|  | *HmGr30* | *HmGr30* | *HmGr30* | *HmGr30* | *H* |  |
|  | *HmGr42* |  |  | *HmGr42* | *H, D* |  |
|  | *HmGr44* | *HmGr44* | *HmGr44* | *HmGr44* | *H, duplicated in B and D* |  |
|  | *HmGr46* | *HmGr46* | *HmGr46* | *HmGr46* | *B, H, duplicated in D* |  |
|  | *HmGr50* | *HmGr50* | *HmGr50* | *HmGr50* | *H* |  |
|  | *HmGr51* | *HmGr51* | *HmGr51* |  | *H* |  |
|  | *HmGr52* | *HmGr52* | *HmGr52* | *HmGr52* | *H, duplicated in D* | sugar |
|  | *HmGr55* | *HmGr55* |  | *HmGr55* | *H* |  |
|  | *HmGr56* | *HmGr56* | *HmGr56* | *HmGr56* | *H* | synephrine related |
|  |  | *HmGr60* | *HmGr60* |  | *H* |  |
|  | *HmGr63* | *HmGr63* | *HmGr63* | *HmGr63* | *B, D, H* | co-receptor |
|  | *HmGr61*^†^*/64/*  *65* | *HmGr61*^†^*/64/65* | *HmGr61*^†^*/64/65* | *Hm61*^†^*/64/*  *65* | *H* |  |
|  | *HmGr66** | *HmGr66* |  | *HmGr66* | *B, D, H* | bitter |
|  |  | *HmGr67* | *HmGr67** |  | *H* |  |
|  | *HmGr70/71* | *HmGr71* |  |  | *H* |  |
|  |  |  |  |  |  |  |
| Male-specific |  |  | *HmGr11** |  | *H, B* |  |
|  | *HmGr25* |  |  |  | *B, D, duplicated in H* |  |
|  |  |  | *HmGr31* |  | *H* |  |
|  | *HmGr69* |  |  |  | *H* |  |
|  |  |  |  |  |  |  |
| Female-specific |  | *HmGr2* |  | *HmGr2* | *B, D, H* | CO_2_ |
|  |  | *HmGr21* |  |  | *H* |  |
|  |  | *HmGr32* |  |  | *H* |  |
|  |  | *HmGr33* |  | *HmGr33* | *H* |  |
|  |  | *HmGr38* |  | *HmGr38* | *H* |  |
|  |  | *HmGr41* |  |  | *H* |  |
|  |  | *HmGr43* |  |  | *H, many duplicates in D* |  |
|  |  | *HmGr45* |  |  | *H, D* | sugar |
|  |  | *HmGr47* |  | *HmGr47* | *H* |  |
|  |  | *HmGr68* |  |  | *B, D, H* |  |
|  |  |  |  | *HmGr54* | *B, D, H* |  |

*B, D, H* indicates the orthologous gene is present in *Bombyx, Danaus* and *Heliconius* genomes, respectively. *H* indicates that the gene is present only in the *H. melpomene* genome.

*Amino acid substitutions in mapped reads compared to reference genome; ^†^Expressed pseudogene.
