# Supplementary material for: Female Behaviour Drives Expression and Evolution of Gustatory Receptors in Butterflies
Source: PLoS Genet. 2013 Jul 11;9(7):e1003620. doi: 10.1371/journal.pgen.1003620 (PMC3732137; doi:10.1371/journal.pgen.1003620)
Supplement: Table S11 — Gustatory receptor mRNAs expressed in adult H. melpomene labial palps and proboscis. (DOC) [file pgen.1003620.s012.doc]

**Table S11. Gustatory receptor mRNAs expressed in adult *H. melpomene* labial palps and proboscis.**

|  | **Male 1** | **Female 1** | **Male 2 & 3** | **Female 2 & 3** | **Lineage** | **Putative Function** |
| --- | --- | --- | --- | --- | --- | --- |
| Both sexes | *HmGr1* | *HmGr1* | *HmGr1* | *HmGr1* | *B, D, H* | CO_2_ |
|  | *HmGr3* | *HmGr3* | *HmGr3* | *HmGr3* | *B, D, H* | CO_2_ |
|  | *HmGr4* | *HmGr4* | *HmGr4* | *HmGr4* | *B, D, H* | sugar |
|  | *HmGr5* | *HmGr5* | *HmGr5* |  | *H, D* | sugar |
|  | *HmGr7** | *HmGr7* | *HmGr7* | *HmGr7* | *B, D, H* | sugar |
|  | *HmGr12** | *HmGr12* | *HmGr12* |  | *H* |  |
|  | *HmGr14* | *HmGr14* | *HmGr14* | *HmGr14* | *H* |  |
|  | *HmGr15* | *HmGr15* | *HmGr15* | *HmGr15* | *H* |  |
|  | *HmGr17* | *HmGr17* | *HmGr17* | *HmGr17* | *Duplicated in B, D, H* |  |
|  | *HmGr19* | *HmGr19* |  |  | *Duplicated in D, H* |  |
|  | *HmGr20** | *HmGr20* | *HmGr20* | *HmGr20* | *Duplicated in D, H* |  |
|  | *HmGr21* | *HmGr21** |  | *HmGr21* | *Duplicated in D, H* |  |
|  | *HmGr25* | *HmGr25* |  | *HmGr25* | *B, D, duplicated in H* |  |
|  | *HmGr30** | *HmGr30* | *HmGr30* | *HmGr30* | *H* |  |
|  | *HmGr22* | *HmGr22* | *HmGr22* | *HmGr22* | *B, D, duplicated in H* |  |
|  | *HmGr33* | *HmGr33* |  |  | *H* |  |
|  | *HmGr34* | *HmGr34* | *HmGr34* |  |  |  |
|  | *HmGr35** | *HmGr35** | *HmGr35* | *HmGr35* | *H* |  |
|  | *HmGr38* | *HmGr38* |  |  | *Duplicated in D, H* |  |
|  | *HmGr39** | *HmGr37/39* | *HmGr39* | *HmGr39* | *Duplicated in D, H* |  |
|  | *HmGr44* | *HmGr44* | *HmGr44* | *HmGr44* | *H, three copies in D* |  |
|  | *HmGr45* | *HmGr45* |  |  | *H, duplicated in D* | sugar |
|  | *HmGr46* | *HmGr46** | *HmGr46** | *HmGr46** | *B, H, duplicated in D* |  |
|  | *HmGr47* | *HmGr47* |  |  | *Duplicated in B, H* |  |
|  | *HmGr50* | *HmGr50* | *HmGr50* | *HmGr50* | *Duplicated in B, H* |  |
|  | *HmGr52* | *HmGr52* | *HmGr52* | *HmGr52* | *H, duplicated in D* | sugar |
|  | *HmGr56* | *HmGr56/57* | *HmGr56* | *HmGr56* | *Duplicated in D, H* | synephrine related |
|  | *HmGr59** | *HmGr59* | *HmGr59* | *HmGr59* | *H* |  |
|  | *HmGr61^†^/64/65* | *HmGr61^†^/64/65* | *HmGr61^†^/64/65* | *HmGr61^†^/64/65* | *H* |  |
|  | *HmGr63* | *HmGr63* | *HmGr63* | *HmGr63* | *B, D, H* | co-receptor |
|  | *HmGr66* | *HmGr66* |  |  | *H, D, duplicated in B* | bitter |
|  | *HmGr67* | *HmGr67** | *HmGr67* | *HmGr67* | *H* |  |
|  | *HmGr68* | *HmGr68* |  |  | *B, D, H* |  |
|  | *HmGr70* | *HmGr70** | *HmGr70* |  | *H* |  |
|  | *HmGr73** | *HmGr73* | *HmGr73* |  | *H* |  |
|  |  |  |  |  |  |  |
| Male-specific | *HmGr11* |  | *HmGr11* |  | *B, H* |  |
|  | *HmGr16* |  |  |  |  |  |
|  | *HmGr31* |  |  |  | *H* |  |
|  | *HmGr32* |  | *HmGr32* |  |  |  |
|  | *HmGr42* |  | *HmGr42* |  | *H, D, duplicated in B* |  |
|  | *HmGr65* |  |  |  |  |  |
|  | *HmGr69* |  |  |  |  |  |
|  | *HmGr71* |  |  |  |  |  |
|  | *HmGr72* |  | *HmGr72* |  | *H* |  |
|  |  |  |  |  |  |  |
| Female-specific |  | *HmGr6* |  | *HmGr6* | *B, D, H* | sugar |
|  |  | *HmGr24* |  |  | *B, D, duplicated in H* |  |
|  |  | *HmGr27* |  | *HmGr27* | *H* |  |
|  |  | *HmGr36* |  |  | *H* |  |
|  |  | *HmGr51* |  |  | *H* |  |
|  |  | *HmGr58* |  |  |  |  |
|  |  | *HmGr60* |  |  | *H* |  |

*B, D, H* indicates the orthologous gene is present in *Bombyx, Danaus* and *Heliconius* genomes, respectively. *H* indicates that the gene is present only in the *H. melpomene* genome.

*Amino acid substitutions in mapped reads compared to reference genome. †Expressed pseudogene.
