# Supplementary material for: Female Behaviour Drives Expression and Evolution of Gustatory Receptors in Butterflies
Source: PLoS Genet. 2013 Jul 11;9(7):e1003620. doi: 10.1371/journal.pgen.1003620 (PMC3732137; doi:10.1371/journal.pgen.1003620)
Supplement: Table S12 — Olfactory receptor mRNAs expressed in adult H. melpomene antennae. (DOC) [file pgen.1003620.s013.doc]

**Table S12.** **Olfactory receptor mRNAs expressed in adult *H. melpomene* antennae.**

| **Tissue** | **Male 1** | **Female 1** | **Male 2 & 3** | **Female 2 & 3** | **Lineage** |
| --- | --- | --- | --- | --- | --- |
| Both sexes | *HmOr2* | *HmOr2* | *HmOr2* | *HmOr2* | *B, D, H* |
|  | *HmOr3* | *HmOr3* | *HmOr3* | *HmOr3* | *H, D* |
|  |  | *HmOr4* | *HmOr4* | *HmOr4* | *H, D* |
|  | *HmOr6* | *HmOr6* | *HmOr6* | *HmOr6* | *H* |
|  | *HmOr7* | *HmOr7* | *HmOr7* | *HmOr7* | *H* |
|  | *HmOr8* | *HmOr8* | *HmOr8* | *HmOr8* | *B, D, H* |
|  | *HmOr9* | *HmOr9* | *HmOr9* | *HmOr9* | *H* |
|  | *HmOr10* | *HmOr10* | *HmOr10* | *HmOr10* | *H* |
|  | *HmOr11* | *HmOr11* | *HmOr11* | *HmOr11* | *H* |
|  | *HmOr12* | *HmOr12* | *HmOr12* | *HmOr12* | *H* |
|  | *HmOr13* | *HmOr13* | *HmOr13* | *HmOr13* | *B, D, H* |
|  | *HmOr14* | *HmOr14* | *HmOr14* | *HmOr14* | *H, B* |
|  | *HmOr16* | *HmOr16* | *HmOr16* | *HmOr16* | *B, D, H* |
|  | *HmOr18* | *HmOr18* | *HmOr18* | *HmOr18* | *B, D, H* |
|  | *HmOr19* | *HmOr19* | *HmOr19* | *HmOr19* | *H, D* |
|  | *HmOr20* |  |  | *HmOr20* | *B, H* |
|  | *HmOr21* | *HmOr21* |  | *HmOr21* | *H, D* |
|  | *HmOr22* | *HmOr22* | *HmOr22* | *HmOr22* | *H, D, duplicated B* |
|  | *HmOr23* | *HmOr23* | *HmOr23* | *HmOr23* | *B, D, H* |
|  | *HmOr24* | *HmOr24* |  | *HmOr24* | *H* |
|  | *HmOr25* | *HmOr25* | *HmOr25* | *HmOr25* | *H, B* |
|  | *HmOr26* | *HmOr26* | *HmOr26* | *HmOr26* | *B, D, H* |
|  | *HmOr27* | *HmOr27* | *HmOr27* | *HmOr27* | *B, D, H* |
|  | *HmOr28* | *HmOr28* | *HmOr28* | *HmOr28* | *B, D, H* |
|  | *HmOr30* | *HmOr30* | *HmOr30* | *HmOr30* | *H* |
|  | *HmOr31* | *HmOr31* |  | *HmOr31* | *H* |
|  | *HmOr33* | *HmOr33* | *HmOr33* | *HmOr33* | *H* |
|  | *HmOr34* | *HmOr34* | *HmOr34* | *HmOr34* | *H* |
|  | *HmOr35* | *HmOr35* | *HmOr35* | *HmOr35* | *H* |
|  | *HmOr36* |  |  | *HmOr36* | *H* |
|  | *HmOr37* | *HmOr37* | *HmOr37* | *HmOr37* | *H* |
|  | *HmOr39* | *HmOr39* |  | *HmOr39* | *H* |
|  | *HmOr40* | *HmOr40* | *HmOr40* | *HmOr40* | *H, duplicated in D* |
|  | *HmOr41* | *HmOr41* | *HmOr41* | *HmOr41* | *B, D, H* |
|  | *HmOr42* | *HmOr42* |  | *HmOr42* | *B, D, H* |
|  | *HmOr43* | *HmOr43* | *HmOr43* | *HmOr43* | *B, D, H* |
|  | *HmOr44* | *HmOr44* | *HmOr44* | *HmOr44* | *B, D, H* |
|  |  | *HmOr45* | *HmOr45* | *HmOr45* | *H* |
|  | *HmOr48* | *HmOr48* |  | *HmOr48* | *H* |
|  | *HmOr49* | *HmOr49* | *HmOr49* | *HmOr49* | *B, D, H* |
|  | *HmOr50* | *HmOr50* | *HmOr50* | *HmOr50* | *B, D, H* |
|  | *HmOr51* | *HmOr51* | *HmOr51* | *HmOr51* | *H* |
|  | *HmOr52* | *HmOr52* | *HmOr52* | *HmOr52* | *B, D, H* |
|  | *HmOr53* | *HmOr53* |  | *HmOr53* | *B, D, H* |
|  | *HmOr54* | *HmOr54* |  | *HmOr54* | *D, H* |
|  | *HmOr55* | *HmOr55* |  | *HmOr55* | *H* |
|  | *HmOr56* | *HmOr56* | *HmOr56* | *HmOr56* | *B, D, H* |
|  | *HmOr57* | *HmOr57* | *HmOr57* | *HmOr57* | *H, D* |
|  | *HmOr58* | *HmOr58* | *HmOr58* | *HmOr58* | *H* |
|  | *HmOr59* | *HmOr59* | *HmOr59* | *HmOr59* | *H* |
|  | *HmOr60* | *HmOr60* | *HmOr60* | *HmOr60* | *B, D, H* |
|  | *HmOr61* | *HmOr61* | *HmOr61* | *HmOr61* | *B, D, H* |
|  | *HmOr63* | *HmOr63* | *HmOr63* | *HmOr63* | *H* |
|  | *HmOr64* | *HmOr64* | *HmOr64* | *HmOr64* | *H* |
|  | *HmOr65* | *HmOr65* | *HmOr65* | *HmOr65* | *H* |
|  | *HmOr67* | *HmOr67* | *HmOr67* | *HmOr67* | *H, D* |
|  | *HmOr69* | *HmOr69* | *HmOr69* | *HmOr69* | *H, B* |
|  | *HmOr71* | *HmOr71* | *HmOr71* | *HmOr71* | *B, D, H* |
|  | *HmOr73* | *HmOr73* | *HmOr73* | *HmOr73* | *H, B* |
|  | *HmOr74* | *HmOr74* | *HmOr74* | *HmOr74* | *H, D* |
| Female-specific |  | *HmOr5* |  | *HmOr5* | *H* |
|  |  | *HmOr32* |  | *HmOr32* | *B, D, H* |
|  |  | *HmOr46* |  | *HmOr46* | *H* |
|  |  | *HmOr47* |  | *HmOr47* |  |
|  |  | *HmOr62* |  | *HmOr62* | *H* |
|  |  | *HmOr70* |  | *HmOr70* | *H, B* |

**Table S10.** Olfactory receptor mRNAs expressed in *H. melpomene* adult antennae.

| **Tissue** | **Male 1** | **Female 1** | **Male 2 & 3** | **Female 2 & 3** | **Lineage** |
| --- | --- | --- | --- | --- | --- |
| Both sexes | *HmOr2* | *HmOr2* | *HmOr2* | *HmOr2* | *B, D, H* |
|  | *HmOr3* | *HmOr3* | *HmOr3* | *HmOr3* | *H, D* |
|  |  | *HmOr4* | *HmOr4* | *HmOr4* | *H, D* |
|  | *HmOr6* | *HmOr6* | *HmOr6* | *HmOr6* | *H* |
|  | *HmOr7* | *HmOr7* | *HmOr7* | *HmOr7* | *H* |
|  | *HmOr8* | *HmOr8* | *HmOr8* | *HmOr8* | *B, D, H* |
|  | *HmOr9* | *HmOr9* | *HmOr9* | *HmOr9* | *H* |
|  | *HmOr10* | *HmOr10* | *HmOr10* | *HmOr10* | *H* |
|  | *HmOr11* | *HmOr11* | *HmOr11* | *HmOr11* | *H* |
|  | *HmOr12* | *HmOr12* | *HmOr12* | *HmOr12* | *H* |
|  | *HmOr13* | *HmOr13* | *HmOr13* | *HmOr13* | *B, D, H* |
|  | *HmOr14* | *HmOr14* | *HmOr14* | *HmOr14* | *H, B* |
|  | *HmOr16* | *HmOr16* | *HmOr16* | *HmOr16* | *B, D, H* |
|  | *HmOr18* | *HmOr18* | *HmOr18* | *HmOr18* | *B, D, H* |
|  | *HmOr19* | *HmOr19* | *HmOr19* | *HmOr19* | *H, D* |
|  | *HmOr20* |  |  | *HmOr20* | *B, H* |
|  | *HmOr21* | *HmOr21* |  | *HmOr21* | *H, D* |
|  | *HmOr22* | *HmOr22* | *HmOr22* | *HmOr22* | *H, D, duplicated B* |
|  | *HmOr23* | *HmOr23* | *HmOr23* | *HmOr23* | *B, D, H* |
|  | *HmOr24* | *HmOr24* |  | *HmOr24* | *H* |
|  | *HmOr25* | *HmOr25* | *HmOr25* | *HmOr25* | *H, B* |
|  | *HmOr26* | *HmOr26* | *HmOr26* | *HmOr26* | *B, D, H* |
|  | *HmOr27* | *HmOr27* | *HmOr27* | *HmOr27* | *B, D, H* |
|  | *HmOr28* | *HmOr28* | *HmOr28* | *HmOr28* | *B, D, H* |
|  | *HmOr30* | *HmOr30* | *HmOr30* | *HmOr30* | *H* |
|  | *HmOr31* | *HmOr31* |  | *HmOr31* | *H* |
|  | *HmOr33* | *HmOr33* | *HmOr33* | *HmOr33* | *H* |
|  | *HmOr34* | *HmOr34* | *HmOr34* | *HmOr34* | *H* |
|  | *HmOr35* | *HmOr35* | *HmOr35* | *HmOr35* | *H* |
|  | *HmOr36* |  |  | *HmOr36* | *H* |
|  | *HmOr37* | *HmOr37* | *HmOr37* | *HmOr37* | *H* |
|  | *HmOr39* | *HmOr39* |  | *HmOr39* | *H* |
|  | *HmOr40* | *HmOr40* | *HmOr40* | *HmOr40* | *H, duplicated in D* |
|  | *HmOr41* | *HmOr41* | *HmOr41* | *HmOr41* | *B, D, H* |
|  | *HmOr42* | *HmOr42* |  | *HmOr42* | *B, D, H* |
|  | *HmOr43* | *HmOr43* | *HmOr43* | *HmOr43* | *B, D, H* |
|  | *HmOr44* | *HmOr44* | *HmOr44* | *HmOr44* | *B, D, H* |
|  |  | *HmOr45* | *HmOr45* | *HmOr45* | *H* |
|  | *HmOr48* | *HmOr48* |  | *HmOr48* | *H* |
|  | *HmOr49* | *HmOr49* | *HmOr49* | *HmOr49* | *B, D, H* |
|  | *HmOr50* | *HmOr50* | *HmOr50* | *HmOr50* | *B, D, H* |
|  | *HmOr51* | *HmOr51* | *HmOr51* | *HmOr51* | *H* |
|  | *HmOr52* | *HmOr52* | *HmOr52* | *HmOr52* | *B, D, H* |
|  | *HmOr53* | *HmOr53* |  | *HmOr53* | *B, D, H* |
|  | *HmOr54* | *HmOr54* |  | *HmOr54* | *D, H* |
|  | *HmOr55* | *HmOr55* |  | *HmOr55* | *H* |
|  | *HmOr56* | *HmOr56* | *HmOr56* | *HmOr56* | *B, D, H* |
|  | *HmOr57* | *HmOr57* | *HmOr57* | *HmOr57* | *H, D* |
|  | *HmOr58* | *HmOr58* | *HmOr58* | *HmOr58* | *H* |
|  | *HmOr59* | *HmOr59* | *HmOr59* | *HmOr59* | *H* |
|  | *HmOr60* | *HmOr60* | *HmOr60* | *HmOr60* | *B, D, H* |
|  | *HmOr61* | *HmOr61* | *HmOr61* | *HmOr61* | *B, D, H* |
|  | *HmOr63* | *HmOr63* | *HmOr63* | *HmOr63* | *H* |
|  | *HmOr64* | *HmOr64* | *HmOr64* | *HmOr64* | *H* |
|  | *HmOr65* | *HmOr65* | *HmOr65* | *HmOr65* | *H* |
|  | *HmOr67* | *HmOr67* | *HmOr67* | *HmOr67* | *H, D* |
|  | *HmOr69* | *HmOr69* | *HmOr69* | *HmOr69* | *H, B* |
|  | *HmOr71* | *HmOr71* | *HmOr71* | *HmOr71* | *B, D, H* |
|  | *HmOr73* | *HmOr73* | *HmOr73* | *HmOr73* | *H, B* |
|  | *HmOr74* | *HmOr74* | *HmOr74* | *HmOr74* | *H, D* |
|  |  |  |  |  |  |
| Female-specific |  | *HmOr5* |  | *HmOr5* | *H* |
|  |  | *HmOr32* |  | *HmOr32* | *B, D, H* |
|  |  | *HmOr46* |  | *HmOr46* | *H* |
|  |  | *HmOr47* |  | *HmOr47* |  |
|  |  | *HmOr62* |  | *HmOr62* | *H* |
|  |  | *HmOr70* |  | *HmOr70* | *H, B* |
