# Supplementary material for: Female Behaviour Drives Expression and Evolution of Gustatory Receptors in Butterflies
Source: PLoS Genet. 2013 Jul 11;9(7):e1003620. doi: 10.1371/journal.pgen.1003620 (PMC3732137; doi:10.1371/journal.pgen.1003620)
Supplement: Text S1 — Identification of H. melpomene homologs of all described insect Gr subfamilies. (DOC) [file pgen.1003620.s016.doc]

**Female behaviour drives expression and evolution of gustatory receptors in butterflies**

**Text S1**

**S1. Identification of *H. melpomene* homologs of all described insect Gr subfamilies**

**S1.1 HmGr63 as a possible lepidopteran gustatory co-receptor**

**S1.2. CO_2_ receptors**

**S1.3. Sugar and inositol receptors**

**S1.4. A fructose receptor**

S1. *Identification and expression profiling of HmGrs in all described insect Gr subfamilies*

We identified members of all described insect Gr subfamilies in the reference genome of *H. melpomene* including putative fructose, sugar, sugar alcohol, CO_2_ and bitter receptors.

S1.1 *A possible role for HmGr63 as a co-receptor for gustatory receptors.*

*HmGr63* was one of the three most abundant *Gr* transcripts found in all libraries. This *Gr* is a member of a conserved gene family of unknown function that includes *DpGr9* and *BmGr63* (Fig. 3)*.* *Grs* function as heterodimers with obligate co-receptors in other insects such as *Drosophila* [1,2,3] and mosquitoes [4]. The widespread expression of *HmGr63* in out chemosensory libraries makes it a good co-receptor candidate for other lepidopteran *Grs,* which would presumably confer the specificity of ligand-binding.

S.1.2. *Putative carbon dioxide receptors are expressed in Heliconius chemosensory tissues*

We found homologues of three conserved CO_2_ receptors in the *H. melpomene* genome, *HmGr1*, *Gr2*, and *Gr3* (Figure 3). Homologues of each of these CO_2_ receptors are found in monarch, silkmoth, red flour beetle, and mosquito genomes, with one homolog (*Gr2*) lost in the drosophilid lineage [5]. Interestingly, whereas all three proteins contribute to CO_2_ reception in *A. gambiae -* although two are sufficient for CO_2_ perception - [6], we found that only two of the *H. melpomene* putative CO_2_ receptors, *HmGr1* and *HmGr3*, were consistently expressed in male and female antennae, mouthparts and legs. Lower levels of expression were found for *HmGr2* in males and female legs and female antennae (Table S9, S10, Supporting Information). In *Drosophila*, CO_2_ receptors are expressed in the antennae and in *A. gambiae*, they are expressed in the maxillary palps. In Lepidoptera, labial palp sensilla in pit organs are known to be CO_2_ sensitive [7] and sensory neurons specific for CO_2_ have been described on these organs [8]. Recently, Jacquin-Joly and colleagues (2012) found expression of two candidate CO_2_ receptors in the antennae of the moth *S. littoralis* [9]. Thus, it seems likely that Lepidoptera may also detect CO_2_ via their antennae.

S.1.3. *Loss of sugar receptors in silkmoth and their expression in Heliconius*

We found 6 putative sugar receptor genes (*HmGr4, Gr5, Gr6, Gr7, Gr45* and *Gr52*) in the reference genome of *H. melpomene* while the genomes of *D. plexippus* and *B. mori* contain 9 (this study) and 4 [10] sugar receptor genes, respectively (Figure 3). The larger number of sugar receptor genes in the monarch genome is due to duplications of *HmGr45* and *Gr52* homologs, while the smaller number in silkmoth appears to be the result of the loss of homologs of *HmGr5* and *Gr52*. Our expression profiling indicates that all six of the putative sugar receptors are expressed in adults either in labial palps/proboscis (*HmGr4, Gr5, Gr6, Gr7, Gr45* and *Gr52*), antennae (*Gr4, Gr45* and *Gr52*) or legs (*Gr4, Gr6, Gr7, Gr45* and *Gr52*), with *HmGr4* and *HmGr52* being the most commonly expressed receptor, followed by *HmGr45* and *HmGr6* (Tables S9-S11, Supporting Information). We note that both *HmGr45* and *HmGr52* are expressed in male and female *H. melpomene* labial palps/proboscis transcriptomes. The loss of these genes in silkmoth may be due perhaps to the fact that silkmoths do not feed as adults, and accordingly, their palps and proboscis are atrophied.

Elsewhere in the *H. melpomene* genome, we found *HmGr8,* a homolog of the sugar alcohol inositol receptor *BmGr8* [11], and we also newly describe a homolog of this gene in the monarch genome, which we named *DpGr55*; however, we found no evidence of expression of *HmGr8* in the adult tissues we profiled, despite behavioural evidence that adult butterflies are sensitive to *chiro-*inositol [12]. This may be due to the generally low levels of expression of *Grs*, but alternatively it is possible that expression of this gene will be found in caterpillars [13] or in other tissues such as gut. Indeed, it is known that Monarch caterpillar lateral and medial maxillary sensilla are sensitive to inositol [13].

S.1.4. *A putative fructose receptor*

We found a homolog of the *B. mori* fructose receptor, *BmGr9*, in the *H. melpomene* genome, which we named *HmGr9.* In *Bombyx*, this gene was found to be expressed in adult male antennae, larval maxilla, labium, thoracic leg and gut [14]. In our data, *HmGr9* was expressed in both male and female *H. melpomene* antennae (Table S10, Supporting Information) and legs (Table S9, Supporting Information). Homologs of this gene are found in *D. melanogaster* (Gr43a), other Lepidoptera [9,15], the honeybee [5], mosquitoes [16,17] and the beetle *Tribolium castaneum*, whose genome contains 10 paralogs.

**References**

1. Jiao Y, Moon SJ, Wang X, Ren Q, Montell C (2008 ) Gr64f is required in combination with other gustatory receptors for sugar detection in *Drosophila*. Curr Biol 18: 1797–1801.

2. Lee Y, Moon SJ, Montell C (2009) Multiple gustatory receptors required for the caffeine response in *Drosophila*. Proc Natl Acad Sci USA 106: 4495-4500.

3. Kwon JY, Dahanukar A, Weiss LA, Carlson JR (2007) The molecular basis of CO_2_ reception in *Drosophila*. Proc Natl Acad Sci USA 104: 3574-3578.

4. Erdelyan CN, Mahood TH, Bader TS, Whyard S (2012) Functional validation of the carbon dioxide receptor genes in *Aedes aegypti* mosquitoes using RNA interference. Insect Mol Biol 21: 119-127.

5. Robertson HM, Wanner KW (2006) The chemoreceptor superfamily in the honey bee, *Apis mellifera:* expansion of the odorant, but not gustatory, receptor family. Genome Res. 16: 1395–1403.

6. Lu T, Qiu YT, Wang G, Kwon JY, Rutzler M, et al. (2007) Odor coding in the maxillary palp of the malaria vector mosquito *Anopheles gambiae*. Curr Biol 17: 1533-1544.

7. Stange G, Stowe S (1999) Carbon-dioxide sensing structures in terrestrial arthropods. Microsc Res Tech 47: 416-427.

8. Bogner F, Boppre M, Ernst KD, Boeckh J (1986) CO_2_ sensitive receptors on labial palps of *Rhodogastria* moths (Lepidoptera: Arctiidae): physiology, fine structure and central projection. J Comp Physiol A 158 741-749.

9. Jacquin-Joly E, Legeai F, Montagne N, Monsempes C, Francois MC, et al. (2012) Candidate chemosensory genes in female antennae of the noctuid moth *Spodoptera littoralis*. Int J Biol Sci 8: 1036-1050.

10. Kent LB, Robertson HM (2009) Evolution of the sugar receptors in insects. BMC Evol Biol 9: 41.

11. Zhang HJ, Anderson AR, Trowell SC, Luo AR, Xiang ZH, et al. (2011) Topological and functional characterization of an insect gustatory receptor. PLoS ONE 6: e24111.

12. Ozaki K, Ryuda M, Yamada A, Utoguchi A, Ishimoto H, et al. (2011) A gustatory receptor involved in host plant recognition for oviposition of a swallowtail butterfly. Nat Commun 2: 542.

13. Dethier VG, Kuch JH (1971) Electrophysiological studies of gustation in lepidoperous larvae. I. Comparative sensitivity to sugars, amino acids, and glycosides. Z vergl Physiologie 72: 343-363.

14. Sato K, Tanaka K, Touhara K (2011) Sugar-regulated cation channel formed by an insect gustatory receptor. Proc Natl Acad Sci USA 108: 11680–11685.

15. Wanner KW, Robertson HM (2008) The gustatory receptor family in the silkworm moth *Bombyx mori* is characterized by a large expansion of a single lineage of putative bitter receptors. Insect Mol Biol 17:621-629.

16. Kent LB, Walden KK, Robertson HM (2008) The Gr family of candidate gustatory and olfactory receptors in the yellow-fever mosquito *Aedes aegypti.* Chem Senses 33: 79–93.

17. Hill CA, Fox AN, Pitts RJ, Kent LB, Tan PL, et al. (2002) G protein-coupled receptors in *Anopheles gambiae*. Science 298: 176-178.
